# Functional specialization of monocot DCL3 and DCL5 proteins through the evolution of the PAZ domain

**DOI:** 10.1093/nar/gkac223

**Published:** 2022-04-05

**Authors:** Shirui Chen, Wei Liu, Masahiro Naganuma, Yukihide Tomari, Hiro-oki Iwakawa

**Affiliations:** Institute for Quantitative Biosciences, The University of Tokyo, Bunkyo-ku, Tokyo 113-0032, Japan; Department of Computational Biology and Medical Sciences, Graduate School of Frontier Sciences, The University of Tokyo, Bunkyo-ku, Tokyo 113-0032, Japan; Institute for Quantitative Biosciences, The University of Tokyo, Bunkyo-ku, Tokyo 113-0032, Japan; Department of Computational Biology and Medical Sciences, Graduate School of Frontier Sciences, The University of Tokyo, Bunkyo-ku, Tokyo 113-0032, Japan; Institute for Quantitative Biosciences, The University of Tokyo, Bunkyo-ku, Tokyo 113-0032, Japan; Institute for Quantitative Biosciences, The University of Tokyo, Bunkyo-ku, Tokyo 113-0032, Japan; Department of Computational Biology and Medical Sciences, Graduate School of Frontier Sciences, The University of Tokyo, Bunkyo-ku, Tokyo 113-0032, Japan; Institute for Quantitative Biosciences, The University of Tokyo, Bunkyo-ku, Tokyo 113-0032, Japan

## Abstract

Monocot DICER-LIKE3 (DCL3) and DCL5 produce distinct 24-nt small interfering RNAs (siRNAs), heterochromatic siRNAs (hc-siRNAs) and phased secondary siRNAs (phasiRNAs), respectively. The former small RNAs are linked to silencing of transposable elements and heterochromatic repeats, and the latter to reproductive processes. It is assumed that these DCLs evolved from an ancient ‘eudicot-type’ DCL3 ancestor, which may have produced both types of siRNAs. However, how functional differentiation was achieved after gene duplication remains elusive. Here, we find that monocot DCL3 and DCL5 exhibit biochemically distinct preferences for 5′ phosphates and 3′ overhangs, consistent with the structural properties of their *in vivo* double-stranded RNA substrates. Importantly, these distinct substrate specificities are determined by the PAZ domains of DCL3 and DCL5, which have accumulated mutations during the course of evolution. These data explain the mechanism by which these DCLs cleave their cognate substrates from a fixed end, ensuring the production of functional siRNAs. Our study also indicates how plants have diversified and optimized RNA silencing mechanisms during evolution.

## INTRODUCTION

Small interfering RNAs (siRNAs) and microRNAs (miRNAs) are critical players in RNA silencing pathways which regulate various biological processes including organismal development and antiviral immunity (1–4). These small RNAs are processed from either long double-stranded RNAs (dsRNAs) or RNAs with hairpin-like structures by specific ribonucleases called Dicer in animals or Dicer-like (DCL) proteins in plants (5,6). These Dicer and DCL proteins are evolutionary conserved multidomain proteins belonging to the RNase III family (6). While mammals have a single Dicer, plants encode multiple DCL proteins that produce different types of small RNAs (7). For example, the genome of the model plant *Arabidopsis thaliana* encodes four DCL proteins, AtDCL1–4 with precise activities. AtDCL1 produces 20 to 22-nucleotide (nt) miRNAs from miRNA precursors with more variable structures compared to animal ones, while AtDCL4 and 2 generate 21 and 22-nt siRNAs from long dsRNA substrates, respectively (7). These small RNAs then regulate protein and mRNA levels through post-transcriptional gene silencing (7). In contrast, AtDCL3 produces heterochromatic 24-nt siRNAs (hc-siRNAs) that form specific RNA-induced silencing complexes (RISCs) with ARGONAUTE4/6 (AGO4/6). RISCs promote sequence-specific DNA methylation and thus transcriptional gene silencing (8). This RNA-directed DNA Methylation (RdDM) process is essential in repressing transposable elements, responding to stresses and maintaining genome integrity (9–11). In short, the evolution of DCL proteins has led to diverse mechanisms that regulate gene expression at different levels.

**Figure 1.**
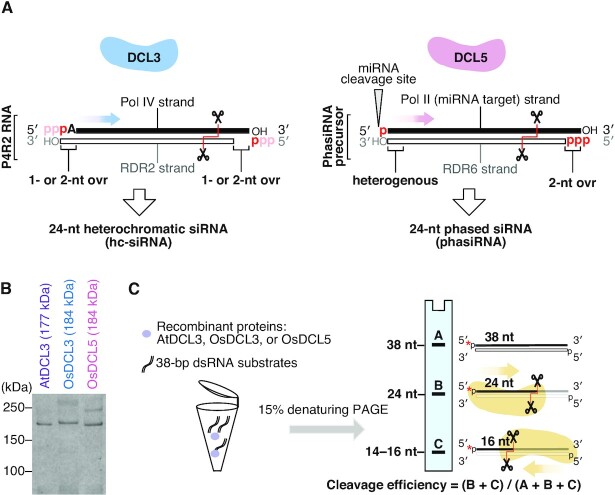
*In vitro* assay determines substrate specificities of DCL3 and DCL5. (**A**) Schematic illustrating *in vivo* substrates for DCL3 and DCL5. The dsRNA substrates for DCL3, named Pol IV and RDR2-dependent RNAs (P4R2 RNAs), have 5′ triphosphates on both strands in theory, which might be converted to monophosphates before cleavage by DCL3. Both strands of the P4R2 precursors hold 1 or 2-nt overhang on the 3′ ends. The cleavage direction is shown with a blue arrow. DCL5 substrates are miR2275 targets, with dsRNA generated by RDR6. Such phasiRNA precursors generally carry heterogenous 3′ structures. The 5′ phosphorylation status of phasiRNA precursors is different on the two strands: with a 5′ monophosphate on one strand (miRNA target strand) and a 5′ triphosphate on the other strand (RDR6 strand). The cleavage direction is shown with a pink arrow. (**B**) Coomassie brilliant blue staining of recombinant AtDCL3, OsDCL3 and OsDCL5 proteins. (**C**) Schematic of *in vitro* dicing assays. dsRNA substrates can be cleaved from the 5′ ends of both strands, mainly generating two cleaved fragments: 24-nt and 14–16-nt. The products of dicing assays were analyzed by denaturing gel. Band A, B and C represent full-length RNA, 24-nt and 14–16-nt products, respectively. Cleavage ratio is calculated by dividing the sum of the cleaved fragments (B + C) by the total amounts of the substrate (A + B + C).

AtDCL3 targets dsRNAs that are generated by the sequential action of two polymerases, DNA-dependent RNA polymerase IV (Pol IV) and RNA-dependent RNA polymerase 2 (RDR2) (12–15). Pol IV synthesizes 30–40-nt RNAs (Pol IV strand), which often bear an adenine at the 5′ end (16,17). RDR2 then synthesizes the complementary strand of the Pol IV strand (RDR2 strand) through its RNA-dependent RNA polymerase activity from the third nucleotide of the Pol IV strand (16,18). The resulting dsRNAs are called Pol IV and RDR2-dependent RNAs (P4R2 RNAs) (19). RDR2 tends to add one or two non-templated nucleotide(s) to the 3′ end of the RDR2 strand via its terminal nucleotidyl transferase activity. Thus, P4R2 RNAs have overhang structures at both ends, typically harboring a 1- or 2-nt 3′ overhang on the RDR2 and Pol IV strands (Figure 1A, left panel) (16,18,19). Interestingly, AtDCL3 preferentially cleaves P4R2 RNAs from the 5′ end of the Pol IV strand (17). This asymmetric dicing by AtDCL3 is thought to be achieved by the combination of 5′ A selection upon Pol IV transcription and preference for the unstable 5′ A and U end resulting from AtDCL3 cleavage (17,20). However, it remains unclear if the 5′ end alone is responsible for the biased cleavage.

A previous study has shown that the recombinant AtDCL3 is capable of cleaving both RNA substrates with 5′ monophosphate and those with 5′ triphosphate, which is a hallmark of nascent transcripts of RNA polymerase (21). On the other hand, it has been reported that the majority of P4R2 RNAs accumulated in *dcl* mutants have 5′ monophosphate instead of 5′ triphosphate (17,19,22). This suggests that the 5′ triphosphate of P4R2 RNA is converted into 5′ monophosphate by an unknown activity. However, it remains unclear whether this conversion is followed by cleavage of P4R2 by DCL3 in wild-type plants.

Some plants produce 24-nt siRNAs that are distinct from hc-siRNAs (23). These siRNAs are called reproductive phased secondary 24-nt siRNAs (24-nt phasiRNA), which are highly expressed in anthers (24,25). Generally, phasiRNAs are produced from the RNAs targeted by 22-nt small RNAs (26–29). The 22-nt small RNA-loaded Argonaute (AGO) proteins cleave the target RNA, resulting in the production of a 3′ cleavage fragment with a 5′ monophosphate. This fragment is then converted into a dsRNA with a triphosphate at the 5′ end of the antisense strand by RDR6, which is recruited via SILENCING DEFECTIVE 5 (SDE5) and the complex consisting of 22-nt small RNA, ARGONAUTE1 (AGO1) and SUPPRESSOR OF GENE SILENCING 3 (SGS3) (30,31). Because RDR6 begins RNA synthesis at the third nucleotide of the template's 3′ end (32), the Pol II (sense) strand of the dsRNA has 2-nt 3′ overhang (Figure 1A, right panel). In contrast, the 3′ end of the RDR6 (antisense) strand of the dsRNA is more heterogeneous, having a blunt end or bearing a 1-nt or 2-nt non-templated nucleotide added by the terminal nucleotidyl transferase activity of RDR6 (Figure 1A, right panel) (32–34). The dsRNA intermediate is then processed by DCLs into phasiRNAs, with the phase determined by the small RNA-guided cleavage site. The mechanism of this one-way processing remains unclear. In monocots, DCL5 (also called DCL3b), which is thought to have evolved via duplication of DCL3, specifically produces 24-nt phasiRNAs in the anther (Supplementary Figure S1) (35). Although eudicots are believed to lack the 24-nt phasiRNA pathway, recent studies argue that some eudicots like *Citrus sinensis* and *Populus trichocarpa* produce 24-nt phasiRNAs even without encoding DCL5 (Supplementary Figure S1) (23,29). In these plants, DCL3 needs to produce both 24-nt phasiRNAs and hc-siRNAs. Given the completely different structures of the dsRNA precursors of 24-nt phasiRNAs and hc-siRNAs, DCL5 and monocot and eudicot DCL3 must have unique substrate specificities to efficiently recognize and cleave their cognate targets. However, so far, the biochemical properties of these DCLs, which are relevant to gene regulation and reproduction, have not been examined and compared.

Dicer and DCL proteins generally consist of five functional domains: the helicase domain, PAZ (PIWI, AGO, and Zwille) domain, two RNase III domains and double-stranded RNA-binding domain from N to C terminal (6,36,37). Previous biochemical and structural studies of human DICER1 and *Drosophila melanogaster* Dicer-2 (Dcr-2) demonstrated that the PAZ domain has two pockets that bind the 5′ and 3′ ends of the substrate dsRNA respectively. These binding pockets are critical for the precise production of small RNAs (38–40). In addition to the PAZ domain, it is reported that the helicase domain interacts with the substrate dsRNA and is required for *Drosophila* Dcr-2 to bind the 3′ end (41–43). Recently, the structures of *Arabidopsis* DCL1 and DCL3 in complex with their substrate dsRNAs have been elucidated (44,45). The structures revealed that the PAZ domain of plant DCLs, like that of animal Dicer, has two pockets that bind to the 5′ and 3′ ends, respectively. However, it is not clear whether the PAZ domain alone is sufficient for the recognition of dsRNA ends. In addition, the substrate recognition mechanisms by other DCLs including DCL5 are so far unknown.

In this study, we succeeded in preparing fully functional recombinant eudicot AtDCL3, monocot *Oryza sativa* DCL3 (OsDCL3) and DCL5 (OsDCL5). Our analysis elucidates how DCL3 and DCL5 have become functionally specialized after gene duplication. OsDCL3 and OsDCL5 have distinct substrate specificities for both 3′ structures and 5′ phosphate, reflecting the different *in vivo* functions of these proteins. Moreover, we find that the PAZ domain is a key determinant of DCL3 and DCL5 substrate specificity. These preferences explain how DCL3 and DCL5 cleave substrates from the fixed end to ensure the production of functional siRNAs. Taken together, our study provides insights into the functional differentiation of DCLs via the evolution of the PAZ domains. This provides a molecular understanding of how plants have diversified and optimized RNA silencing mechanisms through DCL gene duplication.

## MATERIALS AND METHODS

### Plasmid construction

The primers used in this study are listed in Supplementary Table S1.

#### pASW-AtDCL3

AtDCL3 ORF (AT3G43920.3) was amplified from *Arabidopsis thaliana* (Col-0) cDNA using Oligo No. 1 and 2, and cloned into TOPO^®^ vector using pENTR™/D-TOPO™ Cloning Kit (K240020). DCL3 fragment was then introduced into pASW vector using Gateway™ LR Clonase™ II Enzyme mix (Invitrogen™ 11791021). To create the longest AtDCL3 isoform, AT3G43920.2, a 30 bp DNA fragment was inserted into the plasmid by PCR using Oligo No. 3 and 4.

#### pASW-OsDCL3

OsDCL3a (OsDCL3) gene was cloned from the plasmid provided by National Agricultural and Food Research Organization (Clone ID J013008L07), using Oligo No. 5 and 6 and then assembled into pASW vector by NEBuilder Hifi DNA Assembly kit (NEB).

#### pASW-OsDCL5

Four DNA fragments of the OsDCL5 ORF (199–927 nt, 912–2478 nt, 2389–4785 nt and 4786–4914 nt) were amplified from *Oryza sativa* anther cDNA using Oligos No. 7–14. A DNA fragment corresponding to 1–198 nt of OsDCL5 ORF could not be amplified from anther cDNA, and was therefore synthesized by PCR using Oligos No. 15–19 according to the reference sequence of OsDCL3b (OsDCL5) (CDS) from rap-db (The Rice Annotation Project Database) (https://rapdb.dna.affrc.go.jp/viewer/gene_detail/irgsp1?name=Os10t0485600-01;feature_id=339409). These five DNA fragments (1–198 nt, 199–927 nt, 912–2478 nt, 2389–4785 nt and 4786–4914 nt) were assembled into the pASW vector using NEBuilder Hifi DNA Assembly kit (NEB).

#### pASW-OsDCL3_PAZ5

A DNA fragment corresponding to the PAZ domain (2560–3030 nt) of OsDCL5 was amplified by PCR using Oligos No. 20 and 21 from pASW-OsDCL5. Another DNA fragment was amplified by PCR using Oligos No. 22 and 23 from pASW-OsDCL3. These two fragments were mixed and assembled by the NEBuilder Hifi DNA Assembly kit (NEB).

#### pASW-OsDCL5_PAZ3

A DNA fragment corresponding to the PAZ domain (2560–3030 nt) of OsDCL3 was amplified by PCR using Oligos No. 24 and 25 from pASW-OsDCL3. Another DNA fragment was amplified by PCR using Oligos No. 26 and 27 from pASW-OsDCL5. These two fragments were mixed and assembled by the NEBuilder Hifi DNA Assembly kit (NEB).

#### pASW-AtDCL3_PAZ3

A DNA fragment corresponding to the PAZ domain (2560–3030 nt) of OsDCL3 was amplified by PCR using Oligos No. 28 and 29 from pASW-OsDCL3. Another DNA fragment was amplified by PCR using Oligos No. 32 and 33 from pASW-AtDCL3. These two fragments were mixed and assembled by the NEBuilder Hifi DNA Assembly kit (NEB).

#### pASW-AtDCL3_PAZ5

A DNA fragment corresponding to the PAZ domain (2560–3030 nt) of OsDCL5 was amplified by PCR using Oligos No. 30 and 31 from pASW-OsDCL5. Another DNA fragment was amplified by PCR using Oligos No. 32 and 33 from pASW-AtDCL3. These two fragments were mixed and assembled by the NEBuilder Hifi DNA Assembly kit (NEB).

### Cell culture

*Drosophila* Schneider 2 cells (S2 cells) were cultured in Schneider's *Drosophila* Medium (Gibco) supplemented with 10% (v/v) Fetal Bovine Serum (FBS) (Sigma) and antibiotics at 28°C, sealed with parafilm.

### Production of SBP-tagged AtDCL3, OsDCL3, OsDCL5 and PAZ domain chimeric proteins in *Drosophila* S2 cells

S2 cells (1–1.5 × 10^7^ cells/10 cm dish) were transfected with 10 μg pASW plasmids carrying plant DCL3 family genes (pASW-AtDCL3, OsDCL3 or OsDCL5) or PAZ domain chimeric genes (pASW-AtDCL3_PAZ3, AtDCL3_PAZ5, OsDCL3_PAZ5 or OsDCL5_PAZ3) with 20 μl X-tremeGENE™ HP DNA Transfection Reagent (Roche) following manufacturer's instructions. The transfected cells were harvested after 72 hours for lysate preparation.

### Cell lysate preparation

S2 cells were harvested by centrifugation using a swinging-bucket rotor at 1500 × *g* for 3 min at room temperature. The cell pellet was washed by cold PBS (pH 7.4) and was centrifuged at 1500 × *g* at 4°C. The pellets were then weighed and resuspended in equal volumes of Hypotonic buffer [10 mM HEPES–KOH (pH 7.4), 10 mM potassium acetate, 1.5 mM magnesium acetate] containing 5 mM dithiothreitol (DTT) and 1× EDTA-free Complete Protease Inhibitor tablets (Roche) by tapping and inverting the tubes. The suspension was incubated on ice for 15 min, and then mixed thoroughly with a vortex mixer. A cell disruption vessel (Parr Instrument Company) was used to break open the cells. The lysate was clarified by centrifugation at 17 000 × *g* for 20 min at 4°C. The supernatant was flash frozen in liquid nitrogen and immediately stored at −80°C in single-use aliquots.

### Protein purification by Streptavidin beads

Streptavidin Sepharose High Performance beads (GE Healthcare), equivalent to 25% of the lysate by volume, were washed with 1 ml lysis buffer [30 mM HEPES–KOH (pH 7.4), 100 mM potassium acetate, 2 mM magnesium acetate], and then mixed gently with the lysate. The suspension was incubated for 1 hour at 4°C on a rotator and then washed three times with wash buffer (1× lysis buffer containing 800 mM NaCl and 1% (v/v) Triton X-100). The SBP-tagged protein was then eluted with biotin elution buffer (1× lysis buffer, 5 mM DTT, 30% glycerol and 2.5 mM biotin) at 4°C on a rotator for 20 minutes, and the elution step repeated 3 times. The eluates were flash frozen in liquid nitrogen and immediately stored at −80°C in single-use aliquots after adding BSA to a final concentration of 0.2 mg/ml.

### Preparation of radiolabeled dsRNA substrates

The sequences of the sense and antisense RNAs used in this study are shown in Supplementary Figure S2. Single-stranded RNAs with a 5′ hydroxyl group (OH) were synthesized by GeneDesign Inc.(Osaka Japan), while the sense strand RNA with a 5′ triphosphate was synthesized by Bio-Synthesis (Texas, USA). The antisense strand with a 3′ phosphate was radiolabeled by T4 polynucleotide kinase (3' phosphatase minus) (NEB) and [γ-^32^P]ATP. Strands with a 5′ monophosphate were radiolabeled with T4 polynucleotide kinase (Takara) and [γ-^32^P]ATP. The sense and antisense strands were heat-annealed in lysis buffer as previously described (46). The annealed dsRNAs were then separated by electrophoresis on 15% native polyacrylamide gels. The dsRNAs in gel pieces were excised and eluted by soaking in 2× elution buffer [200 mM Tris–HCl (pH 7.5), 2 mM MgCl_2_, 300 mM NaCl, 2% SDS] overnight at room temperature. dsRNAs were mixed with glycogen, precipitated by isopropanol, rinsed with 70% ethanol, and dissolved in lysis buffer.

### Dicing assay

**Figure 2.**
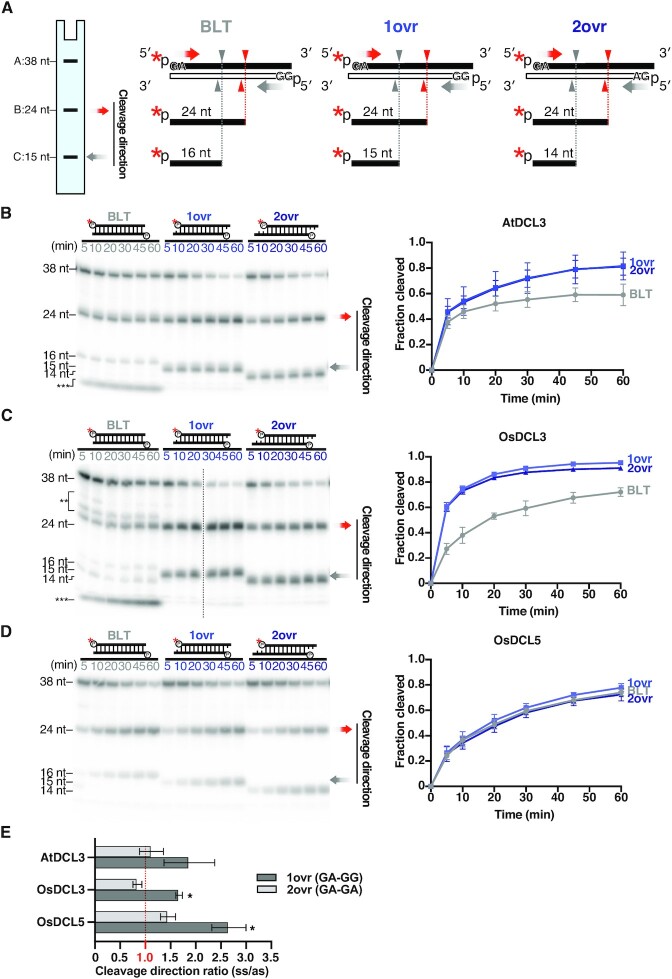
DCL3 and DCL5 have different preferences for 3′ structures. (**A**) 38-nt substrates radiolabeled on the 5′ monophosphate of sense strands, with different 3′ structures: blunt (BLT), 3′ 1-nt overhang (1 ovr) and 3′ 2-nt overhang (2 ovr), were used in the dicing assays (see also Supplementary Figure S2). The red asterisks indicate ^32^P. Red arrowheads and grey arrowheads indicate cleavage from the 5′ end of sense strands and antisense strands, respectively. These substrates can be cleaved into 24-nt (band B) and 14, 15 or 16-nt (band C) products according to their 3′ structures. Cleavage efficiency was calculated as (B + C)/(A + B + C). For BLT, bands of inaccurate cleavage products (long and short fragments) were also calculated as cleavage products. (**B–D**) Left panel: Representative gel images of dicing assays by (B) AtDCL3, (C) OsDCL3 and (D) OsDCL5 cleaving the 38-nt substrates with different 3′ structures (from left to right: BLT, 1ovr and 2ovr). The double and triple asterisks indicate the inaccurate cleavage products, long and short, respectively. Right panel: Quantification of cleavage efficiency in the left panel. The mean values ± SD from three independent experiments are shown. AtDCL3 and OsDCL3 both prefer substrates with 3′ overhangs. OsDCL5 does not show preferences for specific 3′ structures. (**E**) Ratios of cleavage from the 5′ end of sense strand (ss) to that from the antisense strand (as). A ratio of 1 (shown in red) indicates equal cleavage from the 5′ end of sense strand and the antisense strand. When the sense strand and the antisense strand hold 5′ GA and 5′ GG respectively (1ovr), cleavage by all DCL3 family proteins preferred the 5′ GA strand. When both strands hold 5′ GA (2ovr), the ratios are close to 1, suggesting less biased cleavage than 1ovr substrates. Two-tailed paired t-tests with Bonferroni correction were performed to evaluate if these ratios are significantly different from 1, which represents an unbiased cleavage. Asterisks indicate *P* < 0.02 (Supplementary Table S2).

**Figure 3.**
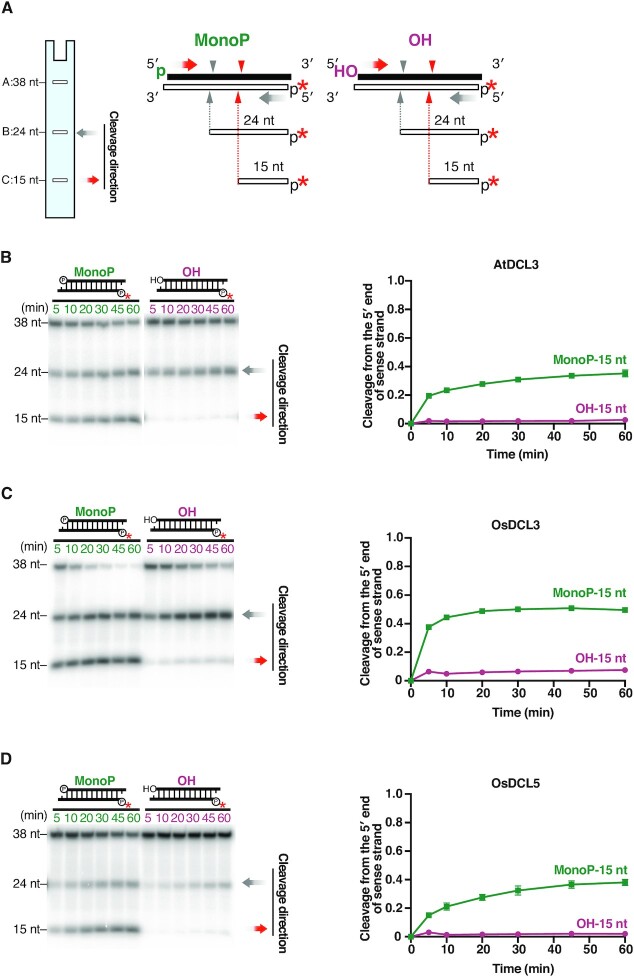
The 5′ monophosphate is important for efficient cleavage by DCL3 and DCL5. (**A**) 1-nt 3′ overhang substrates radiolabeled on the 5′ monophosphate of antisense strands, with a 5′ hydroxyl groups (OH) or a monophosphate (MonoP) on sense strands, were used for dicing assays. The red asterisks indicate ^32^P. Red arrowheads and grey arrowheads indicate cleavage from the 5′ end of sense strands and antisense strands, respectively. Cleavage from the 5′ end of sense strands results in 15-nt products (band C), and the proportion of cleavage from the 5′ end of sense strands is calculated by C/(A + B + C). (**B–D**) Left panel: Representative gel images of dicing assays by (B) AtDCL3, (C) OsDCL3 and (D) OsDCL5 cleaving 1-nt overhang MonoP and OH substrates. Right panel: Quantification of cleavage from the 5′ end of sense strands (15-nt bands) in the left panel. The mean values ± SD from three independent experiments are shown. Compared with a 5′ monophosphate, a 5′ hydroxyl group greatly reduced cleavage from the 5′ end of sense strand by AtDCL3, OsDCL3 and OsDCL5.

**Figure 4.**
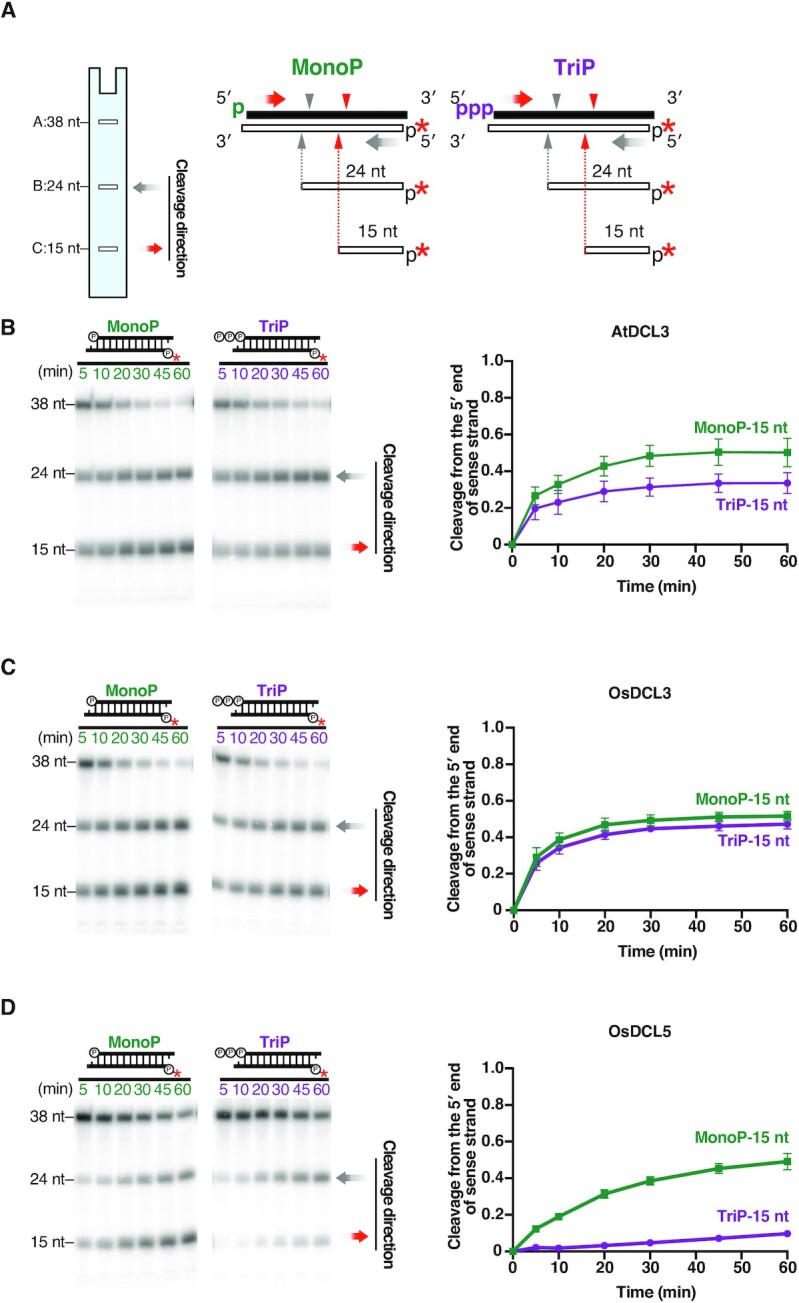
DCL3 and DCL5 have different preferences for the 5′ triphosphate. (**A**) Dicing assays were conducted using 1-nt 3′ overhang substrates radiolabeled on the 5′ monophosphate on the antisense strand, carrying a 5′ monophosphate (MonoP) or triphosphate (TriP) on the sense strands. The red asterisks indicate ^32^P. Red arrowheads and grey arrowheads indicate cleavage from the 5′ end of sense strands and antisense strands, respectively. Cleavage from the 5′ end of sense strands results in 15-nt products (band C), and the proportion of cleavage from the 5′ end of sense strands is calculated as C/(A + B + C). (**B–D**) Left panel: Representative gel image of dicing assays with (B) AtDCL3, (C) OsDCL3 and (D) OsDCL5 cleaving MonoP and TriP substrates. Right panel: Quantification of the proportion of cleavage from the 5′ end of sense strands (15-nt bands) in the left panel. The mean values ± SD from three independent experiments are shown. AtDCL3 slightly prefers substrates with 5′ monophosphate. OsDCL3 does not show an obvious preference for the 5′ mono- or triphosphate. OsDCL5 prefers substrates carrying a 5′ monophosphate.

**Figure 5.**
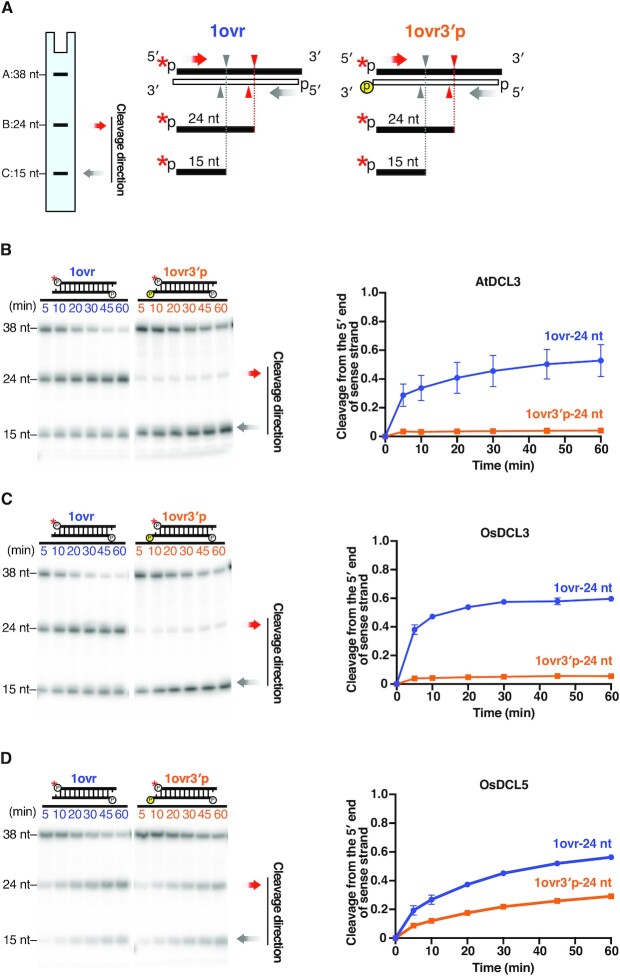
Recognition of the 3′ hydroxyl group is important for DCL3, but not DCL5, cleavage. (**A**) Dicing assays were conducted on 1-nt 3′ overhang substrates radiolabeled on the 5′ monophosphate of sense strands (1ovr). The red asterisks indicate ^32^P. To disrupt recognition of the 3′ hydroxyl, an extra monophosphate group was added to the 3′ end of the antisense strand (1ovr 3′p). Red arrowheads and grey arrowheads indicate cleavage from the 5′ end of sense strands and antisense strands, respectively. Cleavage from 5′ end of sense strands (corresponding to the 3′ monophosphate on antisense strands) results in 24-nt products (band B). (**B–D**) Left panel: Representative gel images of dicing assays by (B) AtDCL3, (C) OsDCL3 and (D) OsDCL5 cleaving 1-nt overhang substrates with or without a 3′ monophosphate on the antisense strand (1ovr 3′p or 1ovr). Right panel: Quantification of the proportion of cleavage from the 5′ end of sense strands (24-nt bands) is shown in the left panel. Mean values ± SD from three independent experiments are shown. The extra 3′ monophosphate greatly reduced cleavage from the 5′ end of sense strand by AtDCL3 and OsDCL3 compared with the 1ovr substrate. Even with the extra 3′ monophosphate (1ovr 3′p), OsDCL5 could still cleave the substrate from the 5′ end of sense strand.

**Figure 6.**
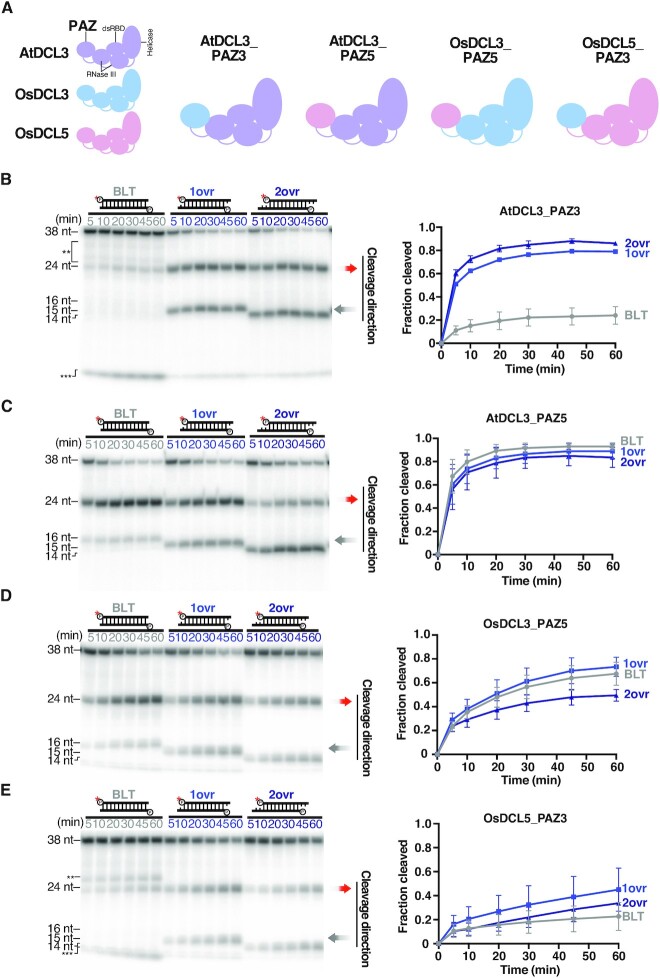
The PAZ domain determines DCL cleavage preferences based on the dsRNA 3′ structure. (**A**) Replacing the PAZ domain of AtDCL3 with PAZ domains of OsDCL3, OsDCL5 generates two chimeric proteins: AtDCL3_PAZ3 and AtDCL3_PAZ5. Swapping the OsDCL3 and OsDCL5 PAZ domains generates two chimeric proteins: OsDCL3_PAZ5 and OsDCL5_PAZ3. (**B–E**) Dicing assays by (B) AtDCL3_PAZ3, (C) AtDCL3_PAZ5, (D) OsDCL3_PAZ5 and (E) OsDCL5_PAZ3 with dsRNA substrates holding different 3′ structures. Left panel: Representative gel images of dicing assays by AtDCL3_PAZ3, AtDCL3_PAZ5, OsDCL3_PAZ5 and OsDCL5_PAZ3 cleaving 38-nt substrates with different 3′ structures (BLT, 1ovr and 2ovr). The double and triple asterisks indicate the inaccurate cleavage products, long and short, respectively. Right panel: Quantification of dicing efficiency in the left panel. For BLT, bands of inaccurate cleavage products (long and short fragments) were also calculated as cleavage products. Mean values ± SD from three independent experiments are shown. AtDCL3_PAZ5 and OsDCL3_PAZ5 can accurately cleave the dsRNA substrates with different 3′ structures, while AtDCL3_PAZ3 and OsDCL3_PAZ3 prefer 3′ overhang over the blunt end structure.

**Figure 7.**
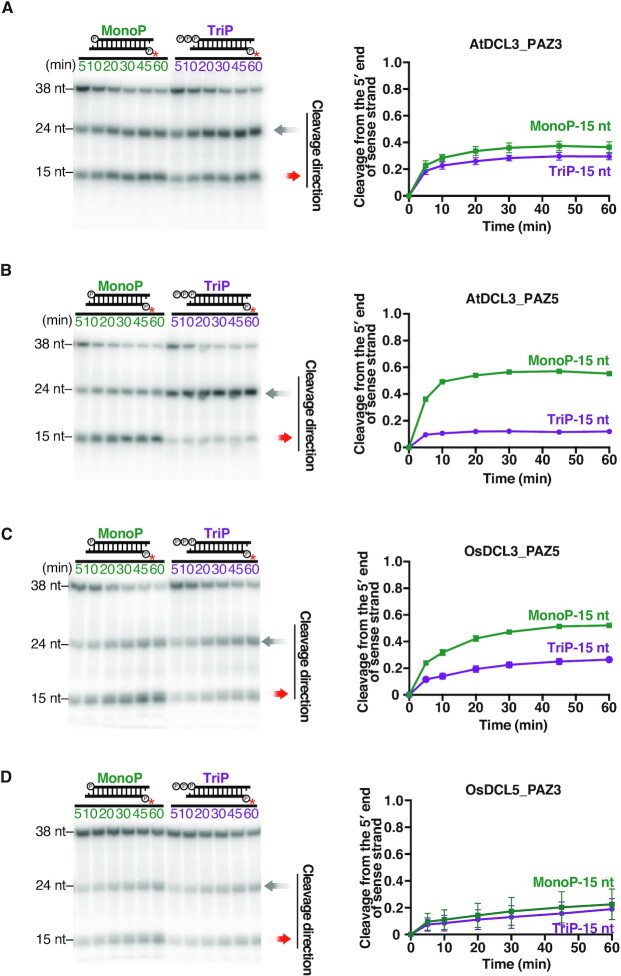
The PAZ domains of DCL5 and DCL3 proteins determines 5′ phosphate preference for dsRNA substrates. Dicing assays by (**A**) AtDCL3_PAZ3, (**B**) AtDCL3_PAZ5, (**C**) OsDCL3_PAZ5 and (**D**) OsDCL5_PAZ3 with MonoP and TriP dsRNA substrates. Left panel: Representative gel images of dicing assays by AtDCL3_PAZ3, AtDCL3_PAZ5, OsDCL3_PAZ5 and OsDCL5_PAZ3 cleaving 38-nt substrates, MonoP and TriP. Right panel: Quantification of the proportion of cleavage from the 5′ end of sense strands (15-nt bands) in the left panel. The mean values ± SD from three independent experiments are shown. AtDCL3_PAZ5 and OsDCL3_PAZ5 prefer 5′ monophosphate, while AtDCL3_PAZ3 and OsDCL5_PAZ3 do not discriminate 5′ monophosphate from triphosphate.

Three nanomolar ^32^P-labeled dsRNAs and 1 nM AtDCL3 or 2 nM OsDCL3, OsDCL5, OsDCL3_PAZ5, OsDCL5_PAZ3, AtDCL3_PAZ3 or 0.25 nM AtDCL3_PAZ5 purified recombinant proteins were incubated in 1× lysis buffer containing 5 mM DTT, 5 mM magnesium acetate, ATP regeneration system [25 mM creatine phosphate (Sigma), 1 mM ATP, 0.03 U/μl creatin kinase (Calbiochem)], and 0.1 U/μl RNasin (Promega) at 25°C. To draw time course curves for each reaction, 2 μl of the reaction mixture was taken at 5,10, 20, 30, 45 and 60 min after the reaction started. These samples were mixed with 8 μl of low-salt PK solution [0.125% SDS, 12.5 mM EDTA, 12.5 mM HEPES–KOH (pH 7.4), 12.5% Proteinase K], and then incubated at 50°C for 10 min. An equal volume of 2× formamide dye [10 mM EDTA (pH 8.0), 98% (w/v) deionized formamide, 0.025% (w/v) xylene cyanol, 0.025% bromophenol blue] was then added and incubated at 95°C for 2 min. The cleavage products were analyzed on 15% denaturing polyacrylamide gels and detected by autoradiography. Small RNA products were quantitated from relative band intensities measured with a Typhoon FLA 7000 image analyzer (GE Healthcare Life Sciences) and quantified using MultiGauge software (Fujifilm Life Sciences). The cleavage efficiency in Figures 2, 6 and Supplementary Figure S3 was calculated by dividing the sum of the cleaved fragments by the total amount of the substrate (full-length + cleaved fragments). For blunt-ended substrates, bands of inaccurate cleavage products (long and short fragments) were also calculated as cleavage products. The cleavage efficiency from the 5′ end of sense strands was calculated by dividing the cleaved 15-nt (Figures 3, 4, 7 and Supplementary Figure S3) or 24-nt (Figure 5 and Supplementary Figure S6) fragments by the total amount of the substrate (full-length + cleaved fragments). The quantitative data and p-values of the triplicate dicing assays are summarized in Supplementary Table S2. Graphs were prepared using GraphPad Prism 8.

## RESULTS

### DCL5 and DCL3 proteins have different preferences for 3′ dsRNA structures

To compare the substrate preferences of monocot DCL5 and monocot and eudicot DCL3 proteins *in vitro*, we successfully prepared full-length recombinant DCL proteins: OsDCL5, OsDCL3 and AtDCL3 using *Drosophila* S2 cells (Figure 1B). Double-stranded RNA (dsRNA) substrates were radiolabeled at the 5′ end of the sense or antisense strand and incubated with purified recombinant DCLs for the *in vitro* dicing assay. Each strand of the substrate was 38 nt long, thus mimicking the length of natural P4R2 RNAs. Since dsRNAs can be cleaved from both ends, two product bands (24-nt and ∼16-nt) were expected (Figure 1C). The cleavage efficiency was calculated by dividing the sum of the cleaved fragments by the total amount of the substrate (full-length + cleaved fragments). To determine the 3′ structures preferred by DCL3 and DCL5 proteins respectively, we performed *in vitro* dicing assays with dsRNAs harboring different 3′ structures: blunt end (BLT), 1-nt overhang (1ovr), and 2-nt overhang (2ovr) (Figure 2A and Supplementary Figure S2). Both AtDCL3 and OsDCL3 cleaved dsRNAs with overhangs more efficiently than the BLT substrates (Figure 2B, C). At early time points (5–30 minutes after incubation), OsDCL3 showed a more pronounced preference for overhangs than AtDCL3 (Figure 2B, C). In contrast, OsDCL5 cleaved BLT, 1ovr and 2ovr dsRNA substrates with similar efficiency (Figure 2D), showing no specific preference for 3′ structures. Interestingly, both AtDCL3 and OsDCL3 generated multiple cleavage products from BLT substrates (Figure 2B, C). We speculate that AtDCL3 and OsDCL3 cannot accurately process BLT substrates, resulting in intermediate products that are longer than 24-nt. These intermediate products might then be cleaved again, generating short fragments observed near the bottom of gels (Figure 2B, C). In contrast, OsDCL5 cleaved BLT as accurately as 1ovr and 2ovr substrates (Figure 2D). We next used a new set of 38-nt dsRNA substrates with different sequence (BLT(B) and OVR(B)) (Supplementary Figures S2 and S3). These results showed a similar trend to that obtained with the original dsRNA substrates (Supplementary Figure S3), suggesting that sequence of dsRNA substrates does not influence the preference for the 3′ structure of dsRNAs in DCL3/5. Taken together, we conclude that DCL5, monocot and eudicot DCL3 proteins have different preferences for the 3′ structure of dsRNAs; OsDCL3 has the strongest preference for 3′ overhangs, followed by AtDCL3, while OsDCL5 has no apparent preference for specific 3′ structures.

A recent structural study showed that the 5′ nucleotide identity and the thermodynamic stability at the terminal base pairs affect the recognition of dsRNA substrates (44). To examine the relationship between the terminal sequence of dsRNA substrates and the cleavage direction, we calculated the ratio of the 24-nt cleavage products to the 15-nt/14-nt cleavage products at the 60 min after the dicing reaction. If the ratio is 1, DCLs cleave the substrates at the same efficiency from both ends. When the ratio is greater than 1, DCLs prefer to cleave the substrates from the 5′ end of the sense strand. On the other hand, if the ratio is less than 1, DCL preferentially cleaves the substrates from the 5′ ends of the antisense strand. Note that only dsRNA substrates with 3′ overhang structures were used in the calculations, because substrates with blunt ends were inaccurately cleaved by AtDCL3 and OsDCL3 (Figure 2B and C and Supplementary Figure S3B and C). By comparing the ratio of 24-nt and 15-nt/14-nt cleavage products from OVR(B) holding 5′ adenine (A) at the sense strand and guanine (G) at the antisense strand (Supplementary Figures S2 and S3A), we found that all the DCLs preferentially cleave the substrates from the 5′ A end (Supplementary Figure S3E). Among them, OsDCL5 has the highest preference for the 5′ A (Supplementary Figure S3E). When both 5′ ends of the dsRNA substrate retained GA (2ovr) (Figure 2A and Supplementary Figure S2), the cleavage direction was not significantly biased in all DCLs (Figure 2E). Interestingly, when the two strands of dsRNA substrates (1ovr) hold 5′ GA and GG respectively (Figure 2A and Supplementary Figure S2), DCL3 and DCL5 preferentially cleaved the substrates from the 5′ GA end (Figure 2E). These results suggest that the thermodynamic stability at the first and the second base pairs from the end of dsRNA substrate contribute to the direction of cleavage.

### The 5′ phosphate of dsRNAs is required for efficient cleavage by both DCL3 and DCL5

In addition to the recognition of the 3′ structure, the recognition of the 5′ phosphate is also important for both accurate and efficient dicing of dsRNAs (41,47). Previous *in vitro* dicing assays using crude plant lysates confirmed that a 5′ phosphate is required for AtDCL3-mediated cleavage of dsRNAs carrying 3′ overhangs (20). To investigate the importance of the 5′ phosphate of dsRNAs in DCL3- and DCL5-mediated cleavage, we performed *in vitro* dicing assays with 3′ 1-nt overhang substrates radiolabeled at the 5′ monophosphate of antisense strands. These substrates carry either a 5′ monophosphate group (MonoP) or a hydroxyl group (OH) on the sense strand (Figure 3A). If the 5′ monophosphate is required for substrate processing, a 5′-hydroxyl should decrease the generation of 15-nt cleavage products which arise from the 5′ end of the sense strand. We found that, for all three DCL proteins, 15-nt products generated from a 5′-OH substrate were decreased compared to MonoP substrates (Figure 3B–D). This result argues that the 5′ phosphate of the substrate is required for efficient dsRNA cleavage by both DCL3 and DCL5.

### DCL3 and DCL5 have distinct preferences for a 5′ triphosphate on the dsRNA

In theory, newly synthesized RNAs generated by Pol IV and RDR2 carry 5′ triphosphates. It is therefore possible that P4R2 RNAs carry a 5′ triphosphate when they encounter DCL3. In addition, precursors of phasiRNAs, i.e. DCL5 substrates, are also likely to possess a triphosphate group at the 5′ end of the antisense strand, which is synthesized by RDR6. To investigate the effect of a 5′ triphosphate group on DCL3 and DCL5-mediated cleavage, we performed *in vitro* dicing assays with dsRNA substrates carrying a 5′-^32^P on the antisense strand. Substrates were monophosphorylated (MonoP) or triphosphorylated (TriP) at the 5′ end of their sense strands (Figure 4A and Supplementary Figure S4A). Since cleavage from the 5′ end of the sense strands results in 15-nt products, the preference for the 5′ phosphate can be quantitated by comparing the proportion of 15-nt bands generated from TriP and MonoP substrates (Figure 4B–D, Supplementary Figure S4B–D). We found that OsDCL5 generated a lower proportion of 15-nt product from TriP compared to MonoP substrates (Figure 4D and Supplementary Figure S4D), indicating that the 5′ triphosphate group strongly inhibits OsDCL5-mediated cleavage. In contrast, the proportion of the 15-nt products cleaved by OsDCL3 was similar for MonoP and TriP substrates (Figure 4C and Supplementary Figure S4C). Thus, the 5′ triphosphate does not affect OsDCL3-mediated dsRNA cleavage. Similarly, we found that AtDCL3 can cleave both MonoP(B) and TriP(B) with equal efficiency (Supplementary Figure S4B). However, when we used another set of MonoP and TriP substrates with different sequences, AtDCL3 generated a lower proportion of 15-nt product from TriP compared to MonoP (Figure 4B). This suggests that AtDCL3 slightly prefers 5′ monophosphate over 5′ triphosphate depending on the substrate sequence. In conclusion, DCL3 and DCL5 proteins have different cleavage efficiencies based on the triphosphate group at the 5′ end of dsRNA, likely impacting the small RNA substrates and pathways they can act upon in plants.

### The PAZ domain determines DCL cleavage preferences based on the dsRNA 3′ structure

Previous studies showed that the PAZ domain of Dicer proteins determines recognition of the 3′ dsRNA structure, in human and *Drosophila* (39,40,48,49). The strong preference of DCL3 for the 3′ overhang prompted us to hypothesize that the interaction between the PAZ domain and the 3′ end of dsRNAs is required for DCL3-mediated dsRNA cleavage. To test this, we introduced an extra phosphate group at the 3′ end of the antisense strand of the 1ovr substrate (1ovr 3′ p) (Figure 5A). This modification is expected to sterically block accommodation of the 3′ overhang by the PAZ domain (Supplementary Figure S5). If the 3′ phosphate inhibits substrate binding to the PAZ domain of DCLs, 24-nt fragments, i.e. cleavage products from the 5′ end of the sense strand, should decrease. In contrast, 15-nt fragments, which represent cleavage from the 5′ end of antisense strand, should increase. Our *in vitro* dicing assays with AtDCL3 or OsDCL3 showed a drastic decrease in the 24-nt fragment and increase in the 15-nt fragment when the 1ovr 3′ p substrate was cleaved. This indicates that 3′ end recognition is important for dicing by AtDCL3 and OsDCL3 (Figure 5B and C). In contrast, although the addition of a 3′ phosphate to the antisense strand decreased the production of the 24-nt fraction, OsDCL5 still cleaved the substrate from the 5′ end of the sense strand (Figure 5D). These data argue that the 3′ end of dsRNA is not strictly recognized by the PAZ domain of DCL5.

To further confirm the importance of the PAZ domain for 3′ recognition, we created chimera AtDCL3s possessing the PAZ domain of OsDCL3 or OsDCL5. We named these chimeric proteins AtDCL3_PAZ3 and AtDCL3_PAZ5 (Figure 6A), and performed *in vitro* dicing assays to investigate their preferences for 3′ structures and 5′ phosphate (Figures 6 and 7). Like OsDCL3, AtDCL3_PAZ3 showed a higher preference for substrates with 3′ overhangs than AtDCL3 (Figures 2B, C and 6B). In contrast, as with OsDCL5, AtDCL3_PAZ5 preferred BLT substrates as well as substrates with 3′ overhangs (Figures 2B, D and 6C). In addition, we observed that AtDCL3_PAZ5 cleaved BLT substrates as accurately as OsDCL5 (Figures 2B, D and 6C), whereas AtDCL3_PAZ3 produced multiple bands, like OsDCL3 (Figures 2B, C and 6B). We also performed dicing assays using substrates with or without an extra 3′ phosphate on the antisense strand (1ovr 3′p vs. 1ovr). As in the case of AtDCL3 and OsDCL3, an extra phosphate added to the 3′ end of the antisense strand significantly inhibited the cleavage by AtDCL3_PAZ3 from the 5′ end of the sense strand. On the other hand, the effect of the extra phosphate was quite mild for AtDCL3_PAZ5, as in the case of OsDCL5 (Supplementary Figure S6A and B). Taken together, we conclude that the PAZ domain plays an important role in determining the preference for 3′ structure and cleavage fidelity of substrates with blunt ends. This conclusion was further supported by experiments with chimeras in which the PAZ domains of OsDCL3 and OsDCL5 were swapped (Figure 6A, D and E and Supplementary Figure S6C and D).

### The PAZ domain in DCL5 and DCL3 proteins determines 5′ phosphate preference on dsRNA substrates

Previous studies have demonstrated that, in human Dicer, several basic amino acid residues in the ‘core’ of the PAZ domain and its upstream Platform domain form a binding pocket for the 5′ end of the dsRNA substrate (40,47). A recent structural study has shown that AtDCL3 also has a similar positively charged 5′-phosphate binding pocket that accommodates the 5′ end of the dsRNA substrate (44). To investigate whether the PAZ domain with the 5′ binding pocket determines the preference for triphosphate, we performed a dicing assay using AtDCL3 mutants with the PAZ domain of OsDCL3 or OsDCL5 (Figures 6A, 7A and B). Strikingly, we found that substitution of the PAZ domain transformed the 5′ phosphate preference of AtDCL3 to that of OsDCL3 or OsDCL5. AtDCL3_PAZ3, a mutant of AtDCL3 with the PAZ domain of OsDCL3, produced similar amounts of 15-nt fragments from the MonoP and TriP substrates (Figure 7A), indicating that, like OsDCL3, AtDCL3_PAZ3 does not discriminate 5′ monophosphate from triphosphate (Figures 4B, C and 7A). In contrast, AtDCL3_PAZ5, a mutant of AtDCL3 with the PAZ domain of OsDCL5, produced less 15-nt fragments from TriP than from MonoP (Figures 4B, D and 7B), indicating that AtDCL3_PAZ5 mimics OsDCL5 and prefers a 5′ monophosphate. Taken together, changing the PAZ domain alters the preference for 5′ triphosphate in DCL3 and DCL5 proteins. This conclusion was further supported by experiments with chimeras that swapped the PAZ domains of OsDCL3 and OsDCL5 (Figures 7C and D). These results suggest that, in addition to the 3′ structure, the PAZ domains of DCL3 and DCL5 determine 5′ phosphate preference during dsRNA cleavage. The PAZ domain therefore plays a key role in determining substrate preferences in plant small RNA-mediated silencing pathways.

## DISCUSSION

### Role of PAZ domains in determining DCL3 family substrate preferences

Previous studies have proposed that substrate preferences of human Dicer and *Drosophila* Dcr-2 proteins are determined by the PAZ domain and helicase domain (38,40,41,47,50). In our study, substitution of the PAZ domain was sufficient to alter substrate preferences for both 3′ structures and 5′ triphosphates for AtDCL3, OsDCL3 and OsDCL5. Our data demonstrate that the PAZ domain alone can determine which dsRNA ends are preferred in DCL3 family proteins. The structures of AtDCL3, recently solved by cryo-EM (44), explain our results that the PAZ domain of DCL3 prefers the 3′ overhang to the blunt end structure. In the complex of AtDCL3 and 1-nt overhang dsRNA substrates, one base pair at the end of the dsRNA is unwound to form 2-nt overhang-like structure, and the 3′ end of dsRNA is recognized by the 3′ binding pocket in the PAZ domain (44). The 2-nt overhang dsRNA is able to bind to the 3′ binding pocket in a similar manner (44). However, in the case of dsRNAs with blunt ends, two base pairs must be unwound in order for the 3′ end to bind to the pocket, which is expected to be energetically disadvantageous compared to the overhang dsRNA substrates. The structure also explains why DCL3 is able to cleave dsRNA substrates from the 5′ triphosphate. In the AtDCL3 and substrate complexes, the 5′ monophosphate is located outside AtDCL3 (44). Thus, even a dsRNA substrate with 5′ triphosphates should be able to bind to DCL3 without collision. On the other hand, since the structure of DCL5 has not been elucidated, it remains unclear why OsDCL5 does not prefer a particular 3′ structure while disliking 5′ triphosphate. Because the important amino acid residues that constitute the 3′ and 5′ binding pockets of DCL3 are essentially conserved in DCL5 (Supplementary Figure S7A and B), the structure of PAZ other than the pockets might determine the specificity for the end of dsRNA substrates. By comparing the amino acid sequences of PAZ domains across DCL3 family proteins, we identified a variable region where DCL3 and DCL5 differ (Supplementary Figure S7C). The corresponding region of human Dicer is located between the 5′ and the 3′ binding pockets in the platform-PAZ cassette. The cassette forms two structurally distinct complexes with short dsRNAs (40); one has a visible α-helix that separates the two pockets, with the 3′ end of the dsRNA anchored in the 3′ pocket and the 5′ end released from the 5′ pocket; the other has a disrupted α-helix that allows anchoring of both ends of the dsRNA in the two pockets. Thus, the α-helix is directly linked to substrate dsRNA binding. In the substrate binding complex of AtDCL3, the corresponding region of the α-helix in human Dicer is disrupted and forms a loop structure, which splits the 5′ end of sense strand and the 3′ end of antisense strand of dsRNA substrates (Supplementary Figure S7D) (44). Given that the amino acid sequences of the loop structure differ significantly between DCL3 and DCL5 (Supplementary Figure S7C), these sequences may impact dsRNA recognition in monocot DCL3 and DCL5. Future structural analysis for the DCL5 should reveal the exact mechanism by which the PAZ domains of DCL3 and DCL5 recognize different substrates.

### Rules for determining the cleavage direction by DCL3

Pol IV collaborates with RDR2 to generate ∼37-nt long dsRNA precursors, P4R2 RNAs, for hc-siRNA production (17). Interestingly, most of the sequenced hc-siRNAs are produced from the 5′ end of the Pol IV strand (17). Currently, this bias is thought to be established by the preference for a 5′ adenine (5′A) at the different steps in the hc-siRNA biogenesis: (i) start site selection by Pol IV (17) and (ii) determination of the dicing direction by DCL3 (20). Our data support these results; when a substrate dsRNA has an A at the 5′ end of sense strand and a G at the 5′ end of the antisense strand, AtDCL3 and OsDCL3 preferentially cleave the dsRNA from the 5′ end of the sense strand with 5′ A (Supplementary Figure S3E). The reason why 5′ A is preferred over 5′ G as a substrate is probably because the A-U base pair with two hydrogen bonds is less stable than the G–C base pair with three hydrogen bonds, making it easier for the ends of substrates to open and bind to the pockets. In addition to the 5′ end nucleotide, we found that the second nucleotide from the 5′ end also affects the biased processing by DCL3. When the substrate with a 5′ GA sense strand and a 5′ GG antisense was used, DCL3s preferentially cleaved the substrate from the 5′ GA end (Figure 2E). In contrast, when dsRNA substrates with 5′ GA at both ends were used, no biased cleavage occurred (Figure 2E). Since neither the second base from the 5′ end of the sense strand nor the opposite base of the antisense strand is recognized by the PAZ domain (44), it is likely that the thermodynamic stability of the second base pair affects the interaction between termini of dsRNA substrate and the corresponding pockets in the PAZ domain by influencing the opening frequency of the 5′ end base pair. The P4R2 RNAs in *Arabidopsis* often possess A and U at the second nucleotide (17). Thus, the thermodynamic stability effect at the second nucleotide may promote biased processing for hc-siRNAs (Supplementary Figure S8A, left panel).

### Preference for a 5′ monophosphate in OsDCL5 determines the direction of cleavage for phased 24-nt siRNA production

Specific miRNAs, including miR390 and 22-nt small RNAs, recruit RDR6 to the target RNA to generate dsRNA precursors (30). This long dsRNA is then processed into phasiRNAs by DCLs (29). Interestingly, DCLs always cleave precursors from the miRNA-mediated cleavage site toward the other end. Although this fixed orientation of dicing is important for production of functional phasiRNAs, how this is achieved has remained unclear. In this study, we found that DCL5, which is known to produce phasiRNAs from RNAs with 22-nt miR2275 target sites, cleaves dsRNAs from a monophosphorylated 5′ end much more efficiently than a tryphosphorylated 5′ end. Since the miRNA-cleaved end has a 5′ monophosphate, whereas the 5′ end of the RDR6 strand has a triphosphate in theory, DCL5 substrate preference explains the directionality of dicing (Supplementary Figure S8A, right panel).

Although *Arabidopsis* does not produce 24-nt phasiRNAs, some eudicots do so even without encoding DCL5 (23,29). In these plants, DCL3 is likely to be responsible for generating 24-nt phasiRNAs. We envision that the slight preference of eudicot DCL3 for 5′ monophosphorylated ends may also contribute to directional processing to produce functional phasiRNAs.

### The preference for 5′ triphosphates in OsDCL3 and AtDCL3 may enhance the production of heterochromatic siRNAs

Since Pol IV uses nucleoside triphosphates (NTPs) as substrates for transcription and lacks binding regions for the capping complexes, Pol IV-synthesized transcripts are expected to possess a triphosphate group at the 5′ end (15,51). RDR2 also generates 5′ triphosphate RNAs *in vitro* (15,16). Thus, nascent P4R2 RNAs theoretically possess 5′ triphosphates at both ends. However, previous studies showed that the P4R2 RNAs that accumulate in the *dcl2/3/4* mutant have monophosphates at the 5′ ends (17,19,22), raising the possibility that unknown RNA phosphatases convert the 5′ triphosphates of dsRNAs into monophosphates in nuclei. In wild-type plants, DCL3 may encounter the P4R2 RNAs before or after the tri- to monophosphate conversion. In any case, OsDCL3’s ability to cleave both 5′ mono- and triphosphorylated dsRNAs with the same efficiency will maximize the production of 24-nt heterochromatic siRNAs (Figure 4C and Supplementary Figures S4C and 8B). Given that AtDCL3 has a slight preference for 5′ monophosphorylated over triphosphorylated precursors in some sequences (Figure 4B and Supplementary Figure S4B), dephosphorylation prior to dicing may enhance the production of a subset of heterochromatic 24-nt siRNAs in *Arabidopsis thaliana* (Supplementary Figure S8B).

### Functional specialization of duplicated DCL3 genes in monocots

**Figure 8.**
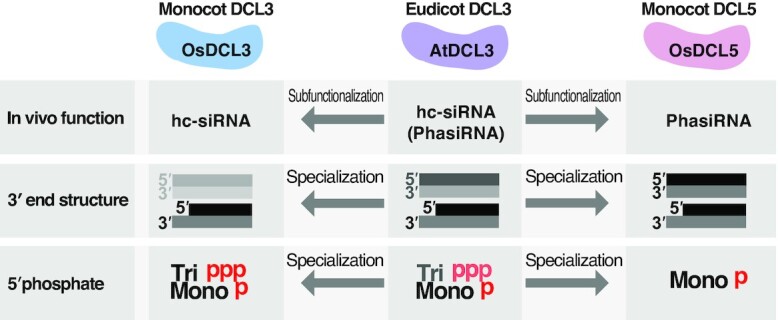
Summary of functions and substrate preferences of DCL3/5 family proteins. Summary of substrate functions and specificities of eudicot DCL3, monocot DCL3 and DCL5. Monocot OsDCL3 and OsDCL5 have completely distinct substrate specificities, whereas eudicot AtDCL3 has an intermediate preference for dsRNAs. These data argue that monocot DCL3 and DCL5 duplicated from a common ancestral ‘eudicot-type’ DCL3, then subfunctionalized and further optimized to cleave cognate substrates.

It is now believed that the appearance of DCL5 in monocots is explained by the ‘sub-functionalization’ of the ancestral DCL3 gene, which is speculated to function in the production of both hc-siRNAs and phasiRNAs (29). However, there is no biochemical evidence supporting this hypothesis. One of the most interesting results in our study may be that monocot OsDCL3 and OsDCL5 have completely different substrate specificities, whereas eudicot AtDCL3 has an intermediate preference for dsRNAs with a 5′ triphosphate and 3′ overhang structure. This implies that monocot DCL5 and DCL3 were not only subfunctionalized, but further optimized for cognate substrates after the duplication from the ancient ‘eudicot-type’ DCL3 (Figure 8). This functional specialization process appears to have been achieved through accumulation of mutations in the PAZ domain. Further biochemical studies on DCLs in a wider variety of plant species will reinforce this hypothesis. Our data, however, indicate how OsDCL family members have evolved to function in specific biological pathways.

## DATA AVAILABILITY

All data are included in the manuscript or in the Supplementary Data and are available from the corresponding author upon request.

## Supplementary Material

gkac223_Supplemental_Files

## Notes

Present address: Masahiro Naganuma, RIKEN Center for Biosystems Dynamics Research, Yokohama, Kanagawa 230-0045, Japan.

Present address: Hiro-oki Iwakawa, Department of Life Science, College of Science, Rikkyo University, Toshima-ku, Tokyo 171-8501, Japan.
