# Supplementary material for: Functional specialization of monocot DCL3 and DCL5 proteins through the evolution of the PAZ domain
Source: Nucleic Acids Res. 2022 Apr 5;50(8):4669–84. doi: 10.1093/nar/gkac223 (PMC9071481; doi:10.1093/nar/gkac223)
Supplement: gkac223_Supplemental_Files [file gkac223_supplemental_files.zip › Supplementary information_final_220402_proof.pdf]

Supplementary information

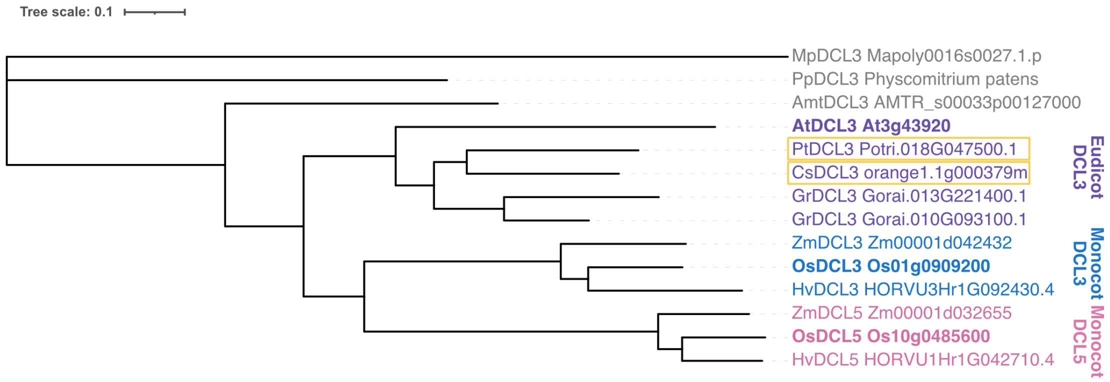

Supplementary Figure 1: Phylogenetic tree of DCL3 family proteins

The phylogenetic tree demonstrates that eudicot DCL3, monocot DCL3 and DCL5 form separate clades. Eudicot plants that produce 24-nt reproductive phasiRNAs are marked with yellow rectangles. *Mp, Marchantia polymorpha*; *Pp*, *Physcomitrium patens*; *Amt*, *Amborella trichopoda*; *At*, *Arabidopsis thaliana*; *Pt*, *Populus trichocarpa*; *Cs*, *Citrus sinensis*; *Gr*, *Gossypium raimondii*; *Os*, *Oryza sativa*; *Zm*, *Zea mays*; *Hv*, *Hordeum vulgare*.

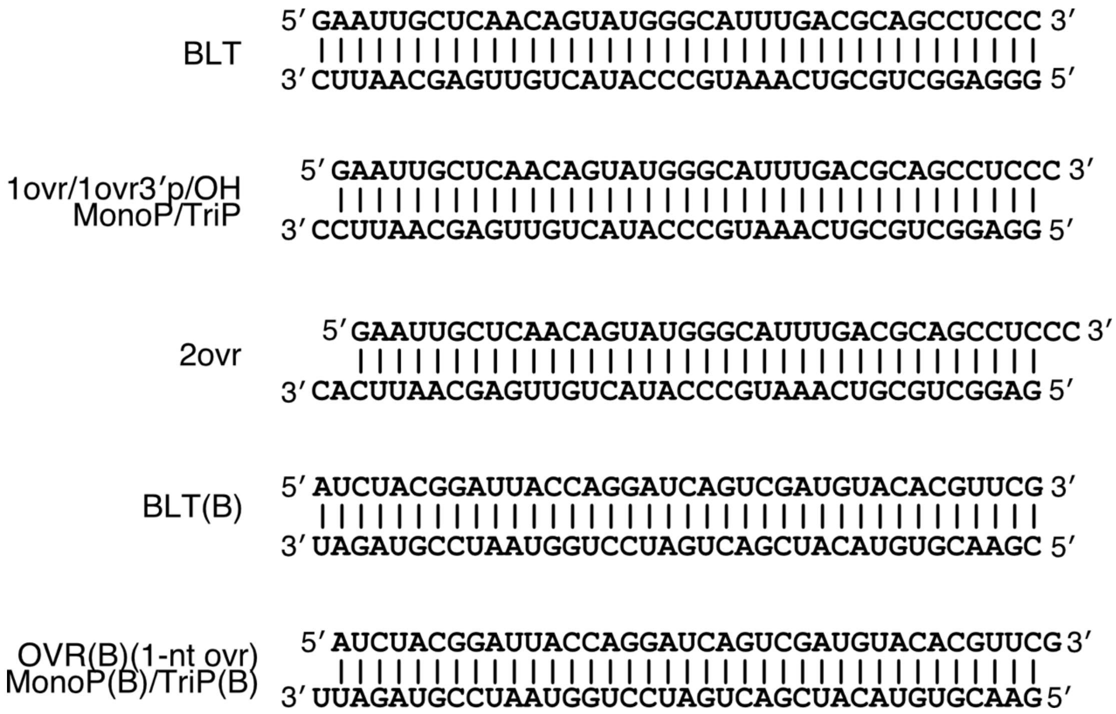

Supplementary Figure 2: RNA sequences used in dicing assays

The sequences and structures of dsRNA substrates used in this study.

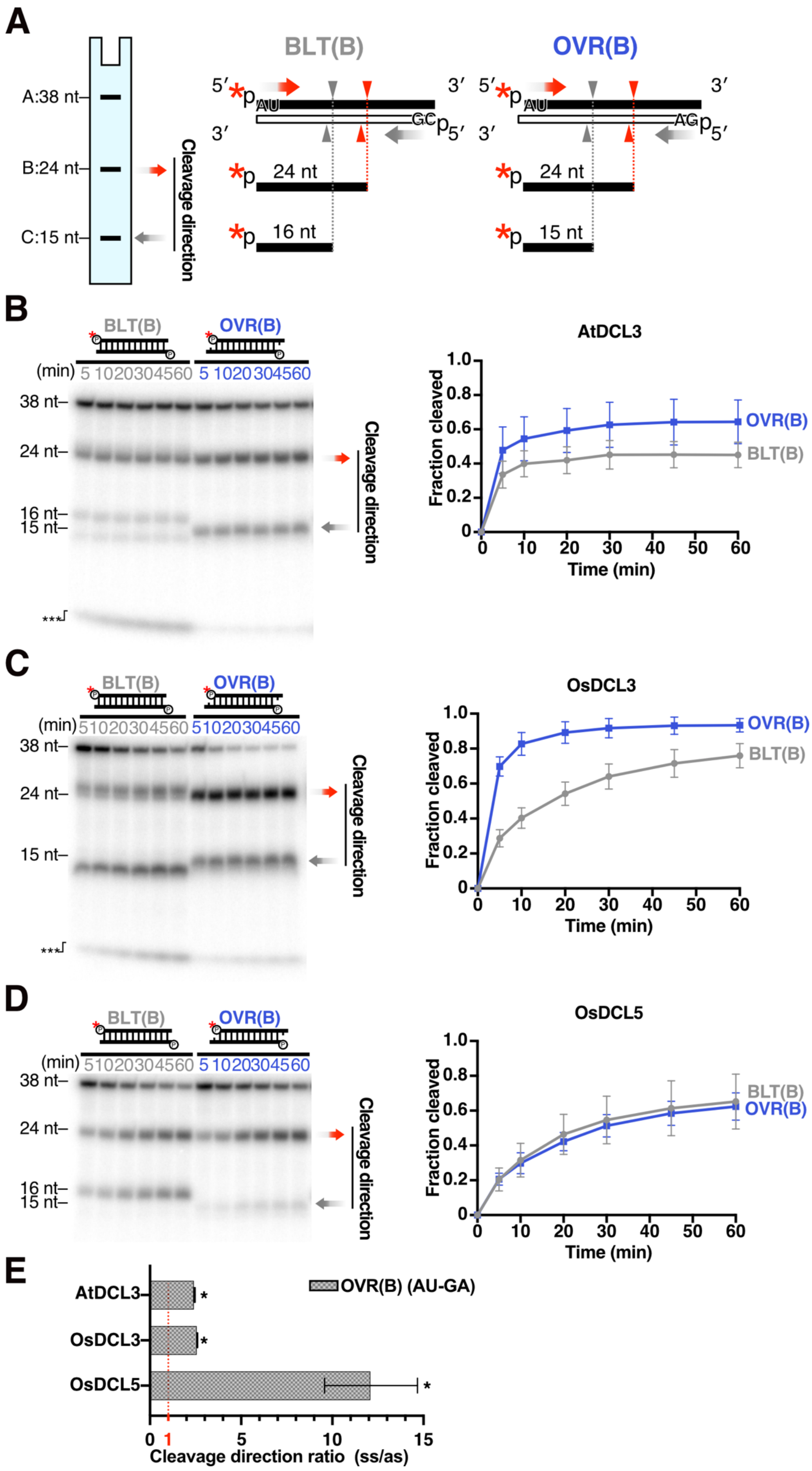
A
A:38 nt
B:24 nt
C:15 nt
Cleavage direction
BLT(B)
OVR(B)
5′
3′
AU
GC
AG
24 nt
16 nt
15 nt
B
(min)
5 10 20 30 45 60
38 nt
24 nt
16 nt
15 nt
***
AtDCL3
Fraction cleaved
Time (min)
C
OsDCL3
D
OsDCL5
E
AtDCL3
OsDCL3
OsDCL5
OVR(B) (AU-GA)
Cleavage direction ratio (ss/as)
0 1 5 10 15

Supplementary Figure 3. DCL3 and DCL5 have different preferences for 3′ structures

(A) New 38-nt substrates radiolabeled on the 5′ monophosphate of sense strands, with 3′ blunt end (BLT(B)) and 3′ 1-nt overhang (OVR(B)) were used in the dicing assays (see also Supplementary Figure 2). The red asterisks indicate ^{32}P. Red arrowheads and grey arrowheads indicate cleavage from the 5′ end of sense strands and antisense strands, respectively. These substrates can be cleaved into 24-nt (band B) and 14,15 or 16-nt (band C) products according to their 3′ structures. Cleavage efficiency is calculated using (B+C)/(A+B+C). For BLT(B), bands of inaccurate cleavage products (long and short fragments) were also calculated as cleavage products.

(B, C, D) Left panel: Representative gel images of dicing assays by (B) AtDCL3, (C) OsDCL3 and (D) OsDCL5 cleaving the 38-nt substrates with different 3′ structures (left panel: BLT(B), right panel: OVR(B)). The double and triple asterisks indicate the inaccurate cleavage products, long and short, respectively. Right panel: Quantification of cleavage efficiency in the left panel. The mean values ± SD from three independent experiments are shown. AtDCL3 and OsDCL3 both prefer substrates with 3′ overhangs. OsDCL5 does not show preferences for specific 3′ structures.

(E) Ratios of cleavage from the 5′ sense strand (ss) to that from the antisense strand (as). A ratio of 1 (shown in red) indicates equal cleavage from the sense strand and the antisense strand. When the sense strand and the antisense strand hold 5′ AU and 5′ GA respectively (OVR(B)), all DCL3 family proteins preferred to cleave from the 5′ AU end, with OsDCL5 showing the most obvious preference. Two-tailed paired t-tests with Bonferroni correction were performed to evaluate if these ratios are significantly different from 1, which represents an unbiased cleavage. Asterisks indicate $p<0.02$ (Supplementary Table 2).

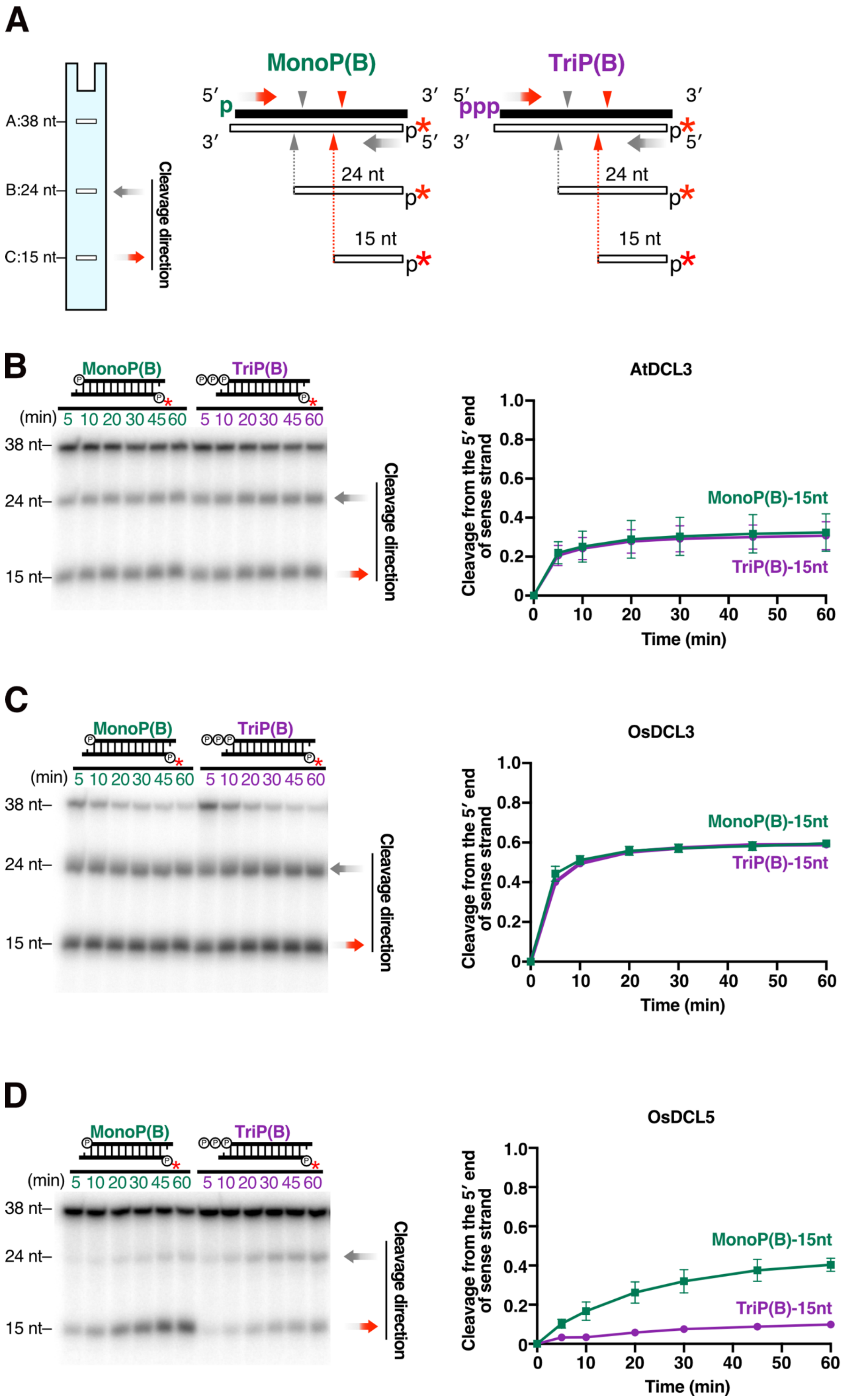

A
A:38 nt
B:24 nt
C:15 nt
Cleavage direction
MonoP(B)
TriP(B)
5′
3′
p
ppp
p
24 nt
15 nt
B
MonoP(B)
TriP(B)
(min) 5 10 20 30 45 60 5 10 20 30 45 60
38 nt
24 nt
15 nt
Cleavage direction
AtDCL3
Cleavage from the 5′ end of sense strand
MonoP(B)-15nt
TriP(B)-15nt
Time (min)
C
MonoP(B)
TriP(B)
(min) 5 10 20 30 45 60 5 10 20 30 45 60
38 nt
24 nt
15 nt
Cleavage direction
OsDCL3
Cleavage from the 5′ end of sense strand
MonoP(B)-15nt
TriP(B)-15nt
Time (min)
D
MonoP(B)
TriP(B)
(min) 5 10 20 30 45 60 5 10 20 30 45 60
38 nt
24 nt
15 nt
Cleavage direction
OsDCL5
Cleavage from the 5′ end of sense strand
MonoP(B)-15nt
TriP(B)-15nt
Time (min)

Supplementary Figure 4. DCL3 and DCL5 have different preferences for the 5′ triphosphate

(A) Dicing assays were conducted using new 1-nt 3′ overhang substrates radiolabeled on the 5′ monophosphate on the antisense strand, carrying a 5′ monophosphate (MonoP(B)) or triphosphate (TriP(B)) on the sense strands. The red asterisks indicate ^{32}P. Red arrowheads and grey arrowheads indicate cleavage from the 5′ end of sense strands and antisense strands, respectively. Cleavage from the 5′ end of sense strands results in 15-nt products (band C), and the proportion of cleavage from the 5′ end of sense strands is calculated as C/(A+B+C).

(B, C, D) Left panel: Representative gel image of dicing assays with (B) AtDCL3, (C) OsDCL3 and (D) OsDCL5 cleaving MonoP(B) and TriP(B) substrates. Right panel: Quantification of the proportion of cleavage from the 5′ end of sense strands (15-nt bands) in the left panel. The mean values ± SD from three independent experiments are shown. AtDCL3 and OsDCL3 do not show an obvious preference for the 5′ mono- or triphosphate. OsDCL5 prefers substrates carrying a 5′ monophosphate.

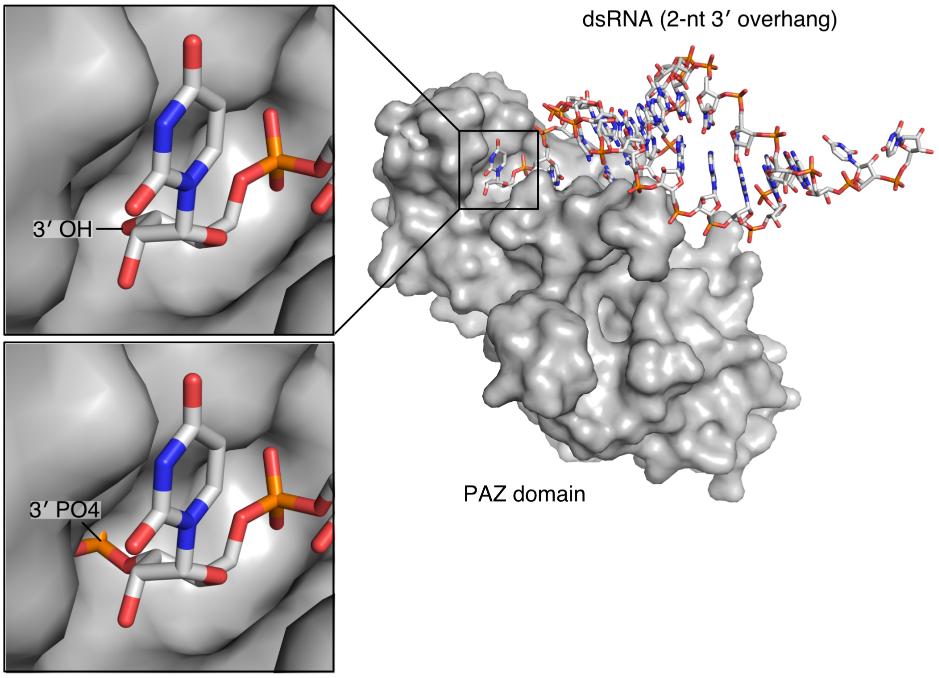

Supplementary Figure 5: An extra 3′ phosphate blocks the binding of 1ovr substrate to the PAZ domain.

Structure of the human Dicer fragment containing the PAZ domain bound to a 2-nt 3′ overhang dsRNA [Protein Data Bank (PDB) ID code: 4ngb]. The 3′ hydroxyl group of the dsRNA substrate is anchored to the 3′ binding pocket (top left). *In silico* replacement of the 3′ hydroxyl group with a 3′ phosphate group results in a steric clash with the 3′ binding pocket (bottom left).

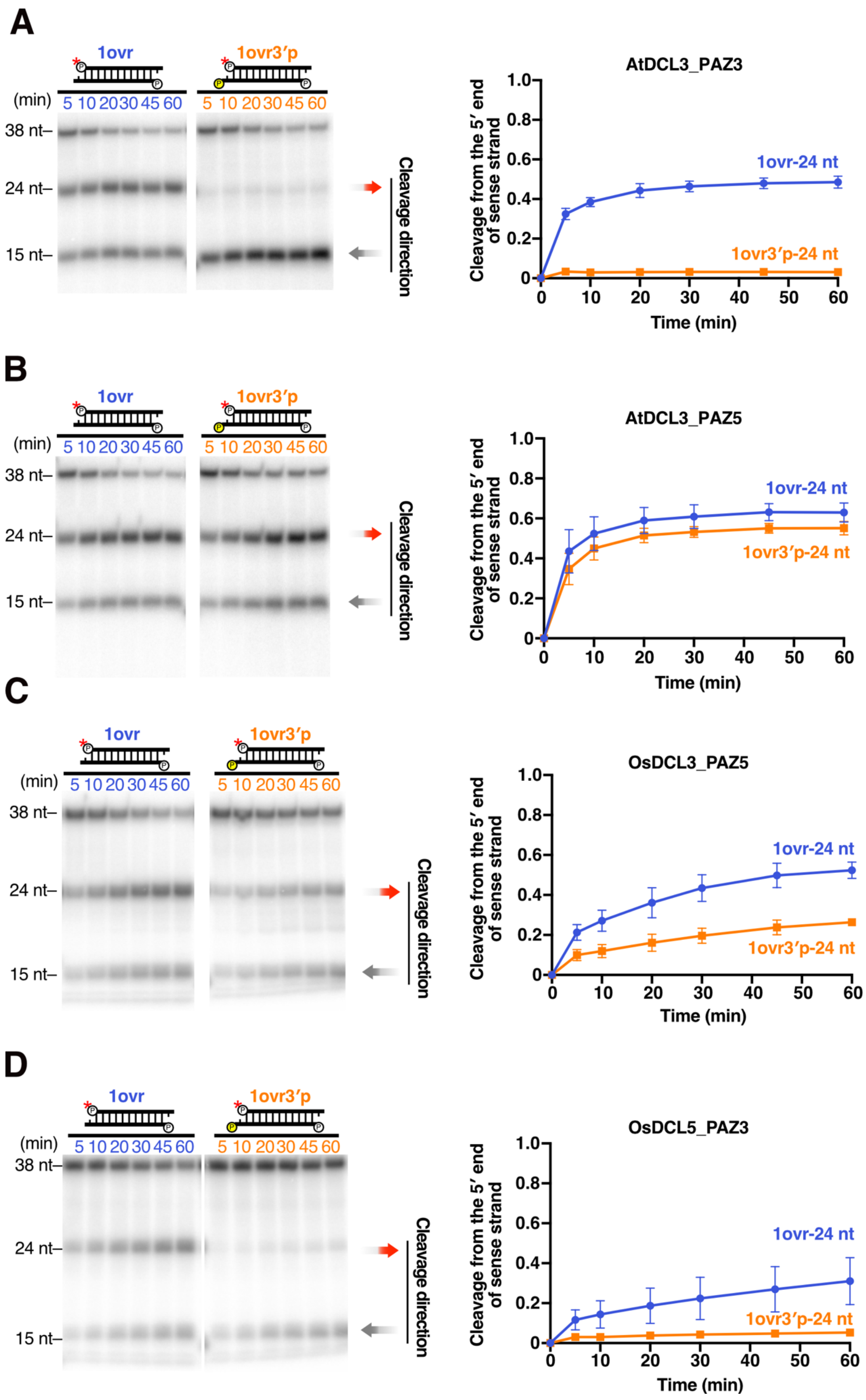
A
B
C
D
1ovr
1ovr3′p
(min)
5 10 20 30 45 60
38 nt–
24 nt–
15 nt–
Cleavage direction
AtDCL3_PAZ3
AtDCL3_PAZ5
OsDCL3_PAZ5
OsDCL5_PAZ3
Cleavage from the 5′ end of sense strand
Time (min)
1ovr-24 nt
1ovr3′p-24 nt
0
0.2
0.4
0.6
0.8
1.0
0 10 20 30 40 50 60

Supplementary Figure 6: Recognition of the 3′ hydroxyl group is critical for cleavage by AtDCL3_PAZ3 and OsDCL5_PAZ3, but not important for cleavage by AtDCL3_PAZ5 and OsDCL3_PAZ5.

(A) Left panel: A representative gel image of AtDCL3_PAZ3 dicing 1-nt overhang substrates with or without a 3′ monophosphate on the antisense strand (1ovr 3′p or 1ovr). Right panel: Quantification of dicing reaction from the 5′ end of sense strands in the left panel. Mean values ± SD from three independent experiments are shown. The extra 3′ phosphate (1ovr 3′p) greatly reduced cleavage from the 5′ end of sense strand by OsDCL3_PAZ3 compared with the 1ovr substrate.

(B) Left panel: A representative gel image of dicing assay by AtDCL3_PAZ5 cleaving the 38-bp substrates 1ovr 3′p and 1ovr. Right panel: Quantification of dicing reaction from the 5′ end of sense strands in the left panel. Mean values ± SD from three independent experiments are shown. Even with the extra 3′ phosphate (1ovr 3′p), AtDCL3_PAZ5 could still cleave the substrate from the 5′ end of sense strand.

(C) Left panel: A representative gel image of OsDCL3_PAZ5 dicing 1-nt overhang substrates with or without a 3′ monophosphate on the antisense strand (1ovr 3′p or 1ovr). Right panel: Quantification of dicing reaction from the 5′ end of sense strands in the left panel. Mean values ± SD from three independent experiments are shown. The extra 3′ phosphate (1ovr 3′p) moderately reduced cleavage from the 5′ end of sense strand by OsDCL3_PAZ5 compared with the 1ovr substrate.

(D) Left panel: A representative gel image of dicing assay by OsDCL5_PAZ3 cleaving the 38-bp substrates 1ovr 3′p and 1ovr. Right panel: Quantification of dicing reaction from the 5′ end of sense strands in the left panel. Mean values ± SD from three independent experiments are shown. Even with the extra 3′ phosphate (1ovr 3′p), OsDCL3_PAZ5 could still cleave the substrate from the 5′ end of sense strand.

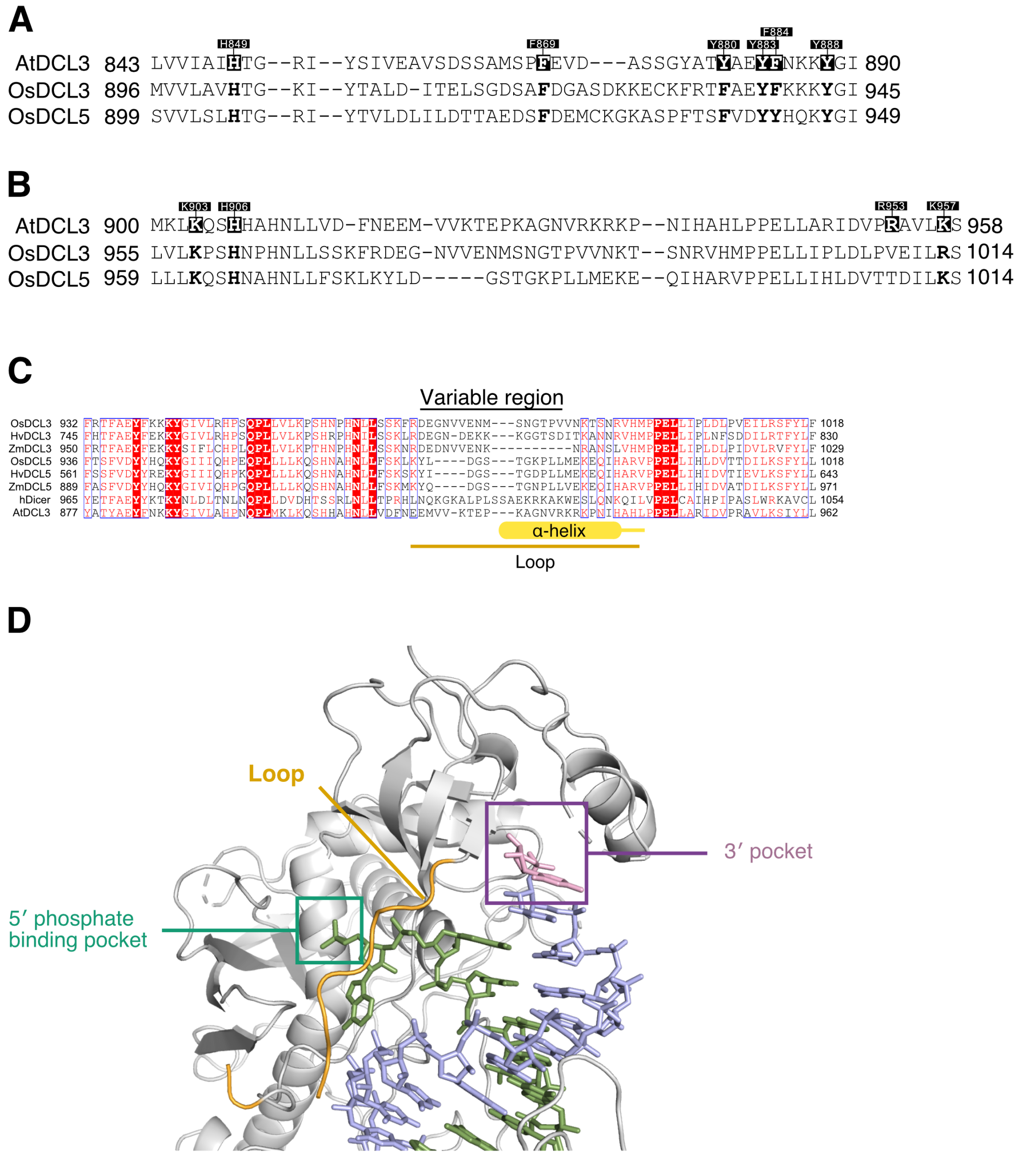

Supplementary Figure 7: Structural insights into the substrate specificities in DCL3 and DCL5.

(A) A multiple sequence alignment of the PAZ domain of *Arabidopsis thaliana* DCL3 (AtDCL3, AT3G43920.3), *Oryza sativa* DCL3 (OsDCL3, Os01g0909200) and OsDCL5 (Os10g0485600). Letters highlighted in black background represents the residues that form the 3′ pocket of AtDCL3. Bold letters indicate the conserved residues in OsDCL3 and OsDCL5 for the 3′ pocket. Note that the AtDCL3 sequence (AT3G43920.3) in the alignment is 10 amino acids shorter than the AT3G43920.2 used in our experiments.

(B) A multiple sequence alignment of the PAZ domain of AtDCL3, OsDCL3 and OsDCL5. Letters highlighted in black background represents the residues that form the 5′ phosphate binding pocket of AtDCL3. Bold letters indicate the conserved residues in OsDCL3 and OsDCL5 for the 5′ phosphate binding pocket.

(C) A multiple sequence alignment of the PAZ domain of human DICER and monocot DCL3 and DCL5. *Os, Oryza sativa*; *Hv*, *Hordeum vulgare*; *Zm*, *Zea mays*. White letters on red background indicate amino acids that are perfectly conserved across human DICER and plant DCL3 family proteins. Red letters indicate amino acids that are highly conserved among human DICER and plant DCL3 family proteins. "α-helix" highlighted in yellow shows the position of the α-helix in the PAZ domain of human DICER, the underline with "Loop" indicates the position of loop structure in the PAZ domain of AtDCL3.

(D) A structure of AtDCL3 fragment containing the PAZ domain bound to a 1-nt 3′ overhang dsRNA (PDB: 7VG2) (1). The loop in the PAZ domain splits the first base pair of the dsRNA substrate, flipping out the 5′ end nucleotide of the sense strand to the 5′ binding pocket. The 3′ end of the antisense strand is recognized by the 3′ binding pocket.

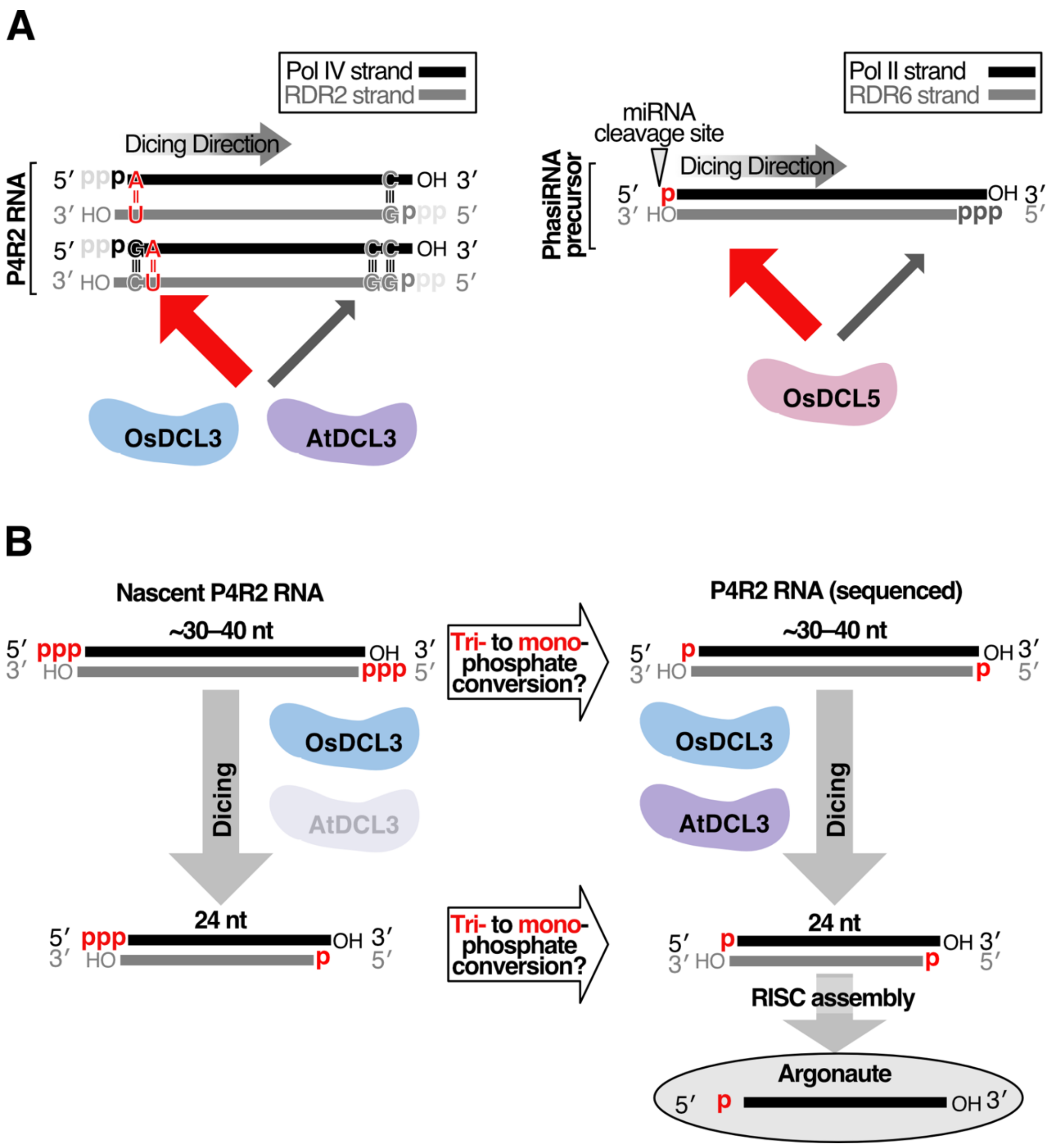

Supplementary Figure 8: Models for unidirectional dicing by DCL3 family members and the order of tri- to monophosphate conversion and dicing by eudicot and monocot DCL3s.

(A) Models for unidirectional dicing mechanisms. AtDCL3 and OsDCL3 cleave P4R2 RNAs from the 5′ end of Pol IV strand as they prefer a 5′A and thermodynamic unstable 5′ end. OsDCL5 cleaves the precursor from the 5′ end of the fragment due to a strong preference for the 5′ monophosphate.

(B) A model for the order of tri- to monophosphate conversion and dicing by eudicot and monocot DCL3s. Since AtDCL3 has a slight preference for 5′ monophosphorylated precursors, dephosphorylation prior to dicing will enhance the production of heterochromatic 24-nt siRNAs in *Arabidopsis thaliana*. In contrast, given that OsDCL3 cleaves both 5′ mono- and triphosphorylated dsRNAs with the same efficiency, OsDCL3 can effectively process P4R2 into 24-nt hc-siRNAs regardless of the tri- to monophosphate conversion.

Supplementary Table 1. The DNA oligos used in this study

Oligo No.	Oligo name	Sequence
1	AtDCL3 F	CACCATGCATTCGTCGTTGGAGCC
2	AtDCL3 R	CTACTTTTGTATTATGACGATCTTGCGGCG
3	AtDCL3 missing F	TTCCTCTCCCTCTAACTATGCAGCTCAAGTATCAGAACTTGAGAGA
4	AtDCL3 missing R	AGTTAGAGGGAGAGGAAACAATGCCTTTTCTAATGACGGCTGACGC
5	OsDCL3 F	GCCGCCCCCTTCACCATGAACCCTTTAAAGAGGTCATTGG
6	OsDCL3 R	CGGCGCGCCCACCCTTTCACACCTCCTGGAGTTGC
7	OsDCL5(199–927) F	CACCATGGTCGCCGTCATGCTC
8	OsDCL5(199–927) R	CCCATGGTATCTGGACAAGCTCTT
9	OsDCL5(912–2418) F	CACCGTCCAGATACCATGGGAAAATATTGTACAG
10	OsDCL5(912–2418) R	CCTTTGTGCTCCACTTTTTCGA
11	OsDCL5(2389–4785) F	CACCCATGGTTCTCGAAAAAGTGGAGCA
12	OsDCL5(2389–4785) R	GTGCAAGGTGATTGTCGAAACAAAG
13	OsDCL5(4786–4914) F	ATACCTGACGCAACAACCATTA
14	OsDCL5(4786–4914) R	CTAAGTCTTGCATATACAGATCTTGAG
15	OsDCL5 1–198_1	ATGGCCGACGACGAGGCTGCCGTCCTCCCGCCCCCGCCTCCGCTGCCGCCGCCTT
16	OsDCL5 1–198_2	GACCCCCTCGGGCGGAGCTGCCTGTGGGGGCGGCAAGGCGGCGGCAGCGGAGGC

17	OsDCL5 1–198_3	TCCGCCCGAGGGGGTCTCGACCGACTGCTGATACCACCCCTCGCACTAGCCAGTT
18	OsDCL5 1–198_4	CGATGGTGTTCCCCCGCAGCGCCGCCTCGAACACCTCCACCAACTGGCTAGTGCGAGGGG
19	OsDCL5 1–198_5	AACACCATCGCGGTGCTCGACACGGGGTCCGGGAAGACC
20	OsDCL5 PAZ F	GCAGCTACGGCAGTTGAGCAGTTGTGGAGTTCGTATCAAG
21	OsDCL5 PAZ R	AGAATGATCTCAAAATATCAGTTGTTACATCGAGATGGATAAGTAGT
22	PAZ exchange OsDCL3 F	ATTTTGAGATCATTCTATTTGTTTCCGGC
23	PAZ exchange OsDCL3 R	AACTGCCGTAGCTGCCG
24	OsDCL3 PAZ F	TTGTGCTGGTGCAGTTAAACTTATGAGAAGGATTTATTCTGAGAATAAAAGAGAAT
25	OsDCL3 PAZ R	AAAATGACTTCAGAATTTCCACAGGTAAATCAAGGGGAATCA
26	PAZ exchange OsDCL5 F	ATTCTGAAGTCATTTTATTTACTCCCTTCTGTAATAC
27	PAZ exchange OsDCL5 R	AACTGCACCAGCACAAGATTCAAT
28	OsDCL3 PAZ F (At)	CTGTGCCTCAATAGTTAAACTTATGAGAAGGATTTATTCTGAGAATAAAAGAGAAT
29	OsDCL3 PAZ R (At)	AGATTGATTTTAGCACTTCCACAGGTAAATCAAGGGGAATCA
30	OsDCL5 PAZ F (At)	CTGTGCCTCAATAGTTGAGCAGTTGTGGAGTTCGTATCAAG
31	OsDCL5 PAZ R (At)	AGATTGATTTTAGCACATCAGTTGTTACATCGAGATGGATAAGTAGT
32	AtDCL3 PAZ exchange F	GTGCTAAAATCAATCTACTTGCTGCCTTC
33	AtDCL3 PAZ exchange R	AACTATTGAGGCACAGGAGTTGATAGC

Supplementary Methods

Evolutionary analysis by Maximum Likelihood method

Evolutionary history was inferred using the Maximum Likelihood method and JTT matrix-based model(2). The tree with the highest log likelihood (-34135.57) is shown. Initial tree(s) for the heuristic search were obtained automatically by applying Neighbor-Join and BioNJ algorithms to a matrix of pairwise distances estimated using the JTT model, and then selecting the topology with superior log likelihood value. The tree is drawn to scale, with branch lengths measured in the number of substitutions per site. This analysis involved 14 amino acid sequences. There were a total of 1843 positions in the final dataset. Amino acid alignment by MUSCLE and evolutionary analyses were conducted in MEGA X(3, 4). Phylogenetic tree was modified using iTOL (https://itol.embl.de)(5).

Multiple alignment of the PAZ domains of animal Dicer and plant DCL3 family proteins

Multiple alignment of the PAZ domains of animal Dicer and plant DCL3 family proteins was performed using the PROMALS3D multiple sequence and structure alignment server(6).

Protein Structure Visualization

Protein Structure Visualization for the platform-PAZ-connector cassette of human Dicer (PDB ID: 4NGB) and AtDCL3 (PDB ID: 7VG2) was performed by PyMol, and *in silico* replacement of the 3′ hydroxyl group on the dsRNA substrate in platform-PAZ-connector cassette (PDB ID: 4NGB) with 3′ phosphate group was done by Coot(7).

Preparation of radiolabeled dsRNA substrates

The sequences of new sense and antisense RNAs used in experiments are shown in supplementary Figure 2. Single-stranded RNAs with a 5′ hydroxyl group (OH) were synthesized by GeneDesign Inc.(Osaka Japan), while the sense strand RNA with a 5′ triphosphate was synthesized by Bio-Synthesis (Texas, USA). The antisense strand with a 3′ phosphate was radiolabeled by T4 Polynucleotide Kinase (3' phosphatase minus) (NEB) and [γ-^{32}P]ATP. Strands with a 5′ monophosphate were radiolabeled with T4 polynucleotide kinase (Takara) and [γ-^{32}P]ATP. The sense and antisense strands were heat-annealed in lysis buffer as previously described (8). The annealed dsRNAs were then separated by electrophoresis on 15% native polyacrylamide gels. The dsRNAs in gel pieces were excised and eluted by soaking in 2 × elution buffer [200 mM Tris-HCl (pH 7.5), 2 mM $MgCl_2$, 300 mM NaCl, 2% SDS] overnight at room temperature. dsRNAs were mixed with glycogen and precipitated by isopropanol, then dissolved in lysis buffer.

Supplementary References

1. Wang,Q., Xue,Y., Zhang,L., Zhong,Z., Feng,S., Wang,C., Xiao,L., Yang,Z., Harris,C.J., Wu,Z., *et al.* (2021) Mechanism of siRNA production by a plant Dicer-RNA complex in dicing-competent conformation. *Science*, **374**, 1152–1157.
2. Jones,D.T., Taylor,W.R. and Thornton,J.M. (1992) The rapid generation of mutation data matrices from protein sequences. *Comput Appl Biosci*, **8**, 275–282.
3. Kumar,S., Stecher,G., Li,M., Knyaz,C. and Tamura,K. (2018) MEGA X: Molecular Evolutionary Genetics Analysis across Computing Platforms. *Mol Biol Evol*, **35**, 1547–1549.
4. Stecher,G., Tamura,K. and Kumar,S. (2020) Molecular Evolutionary Genetics Analysis (MEGA) for macOS. *Mol Biol Evol*, **37**, 1237–1239.
5. Letunic,I. and Bork,P. (2021) Interactive Tree Of Life (iTOL) v5: an online tool for phylogenetic tree display and annotation. *Nucleic Acids Research*, 10.1093/nar/gkab301.
6. Pei,J., Kim,B.-H. and Grishin,N.V. (2008) PROMALS3D: a tool for multiple protein sequence and structure alignments. *Nucleic Acids Res*, **36**, 2295–2300.
7. Emsley,P., Lohkamp,B., Scott,W.G. and Cowtan,K. (2010) Features and development of Coot. *Acta Crystallogr D Biol Crystallogr*, **66**, 486–501.
8. Tomari,Y. and Iwakawa,H.-O. (2017) In Vitro Analysis of ARGONAUTE-Mediated Target Cleavage and Translational Repression in Plants. *Methods Mol Biol*, **1640**, 55–71.
